# When Morphology and Biogeography Approximate Nuclear ITS but Conflict with Plastid Phylogeny: Phylogeography of the *Lotus dorycnium* Species Complex (Leguminosae)

**DOI:** 10.3390/plants11030410

**Published:** 2022-02-02

**Authors:** Tatiana E. Kramina, Maya V. Lysova, Tahir H. Samigullin, Mehmet U. Özbek, Dmitry D. Sokoloff

**Affiliations:** 1 Department of Higher Plants, Biological Faculty, Lomonosov Moscow State University, GSP-1, Leninskie Gory, 119234 Moscow, Russia; dmitry.sokoloff@msu-botany.ru; 2 LLC “Amplitech”, 1-ya Kuryanovskaya Str., 34-8, 109235 Moscow, Russia; m.lysova@amplitech.ru; 3 A.N. Belozersky Institute of Physico-Chemical Biology, Lomonosov Moscow State University, GSP-1, Leninskie Gory, 119991 Moscow, Russia; samigul@belozersky.msu.ru; 4 Department of Biology, Faculty of Science, Gazi University, Teknikokullar, Ankara 06500, Turkey; ufukozbek@gazi.edu.tr

**Keywords:** *Lotus dorycnium*, *Dorycnium pentaphyllum*, *Lotus hirsutus*, nrITS, *trn*L-F, *rps*16, *psb*A-*trn*H, phylogeny, phylogeography, Mediterranean

## Abstract

*Lotus dorycnium* s.l. is a complex of taxa traditionally regarded as members of *Dorycnium*. It has a wide Mediterranean range, extending in the north to Central and Eastern Europe, and in the east to the Crimea, the Caucasus, and the Western Caspian region. Molecular phylogenetic data support placement of the *L. dorycnium* complex in the genus *Lotus*. The present study investigated the phylogeny, phylogeography and morphological variability of the *L. dorycnium* complex across its distribution range to reveal the main trends in genetic and morphological differentiation in this group. The results of the morphological analyses demonstrated some degree of differentiation, with *L. d.* ssp. *herbaceus*, ssp. *gracilis*, and ssp. *anatolicus* more or less well defined, whereas ssp. *dorycnium*, ssp. *germanicus*, and ssp. *haussknechtii* can be hardly distinguished from each other using morphology. Analyses of the *L. dorycnium* complex based on nrITS revealed a tendency towards a geographic differentiation into Western, Eastern, and Turkish groups. Phylogenetic and phylogeographic analyses of the same set of specimens using concatenated plastid markers *trn*L-F, *rps*16, and *psb*A-*trn*H demonstrated a low resolution between the *L. dorycnium* complex and *L. hirsutus*, as well as among the taxa within the *L. dorycnium* complex, which can be interpreted as evidence of an incomplete lineage sorting or hybridization. The evolutionary processes responsible for incongruence in phylogenetic signals between plastid and nuclear sequences of the morphologically well-defined species *L. dorycnium* and *L. hirsutus* were most likely localized in the Eastern Mediterranean. A possibility of rare gene exchange between the *L. dorycnium* complex and the group of *L. graecus* is revealed for the first time.

## 1. Introduction

*Lotus* is the largest and most taxonomically complicated genus of the tribe Loteae (Papilionoideae-Leguminosae). *Dorycnium* Mill. was traditionally accepted as a distinct genus by European botanists [1,2], but on the global scale it cannot be properly separated from *Lotus* in terms of morphology [3,4,5,6]. Molecular phylogenetic data showed that even at the European scale separation of *Dorycnium* is strongly problematic [7,8]. In a monograph of *Dorycnium* which was published 120 years ago but which still remains the latest detailed worldwide study of the group, Rikli [9] recognized three sections within the genus: *Canaria* Rikli, *Bonjeanea* Taubert, and *Eudorycnium* Boiss. (the valid name of the latter section is *Dorycnium*). Members of the sections *Canaria* and *Bonjeanea* combine morphological characters of the genera *Lotus* and *Dorycnium* in their traditional circumscriptions [8]. In the present paper, we follow a wide concept of the genus *Lotus*, which includes all members traditionally classified in sections *Canaria*, *Bonjeanea*, and *Dorycnium*. The section *Canaria* is not closely related to members of the other two sections [7,8,10]. This agrees with earlier ideas of Gillett [4]. *Lotus* section *Bonjeanea*, according to [10], includes *L. rectus* L., *L. strictus* Fisch. & C.A.Mey. and *L. hirsutus* L. The phylogeny and phylogeography of these three species as well as their relatives, such as *L. graecus* L. and two Turkish endemics traditionally classified in the section *Dorycnium*, were studied by Kramina et al. [8].

The present study is devoted to the phylogeny and phylogeography of the *Lotus dorycnium* L. (=*Dorycnium pentaphyllum* Scop.) complex. We consider all members of this group within the genus *Lotus*. The *L.* *dorycnium* complex has a wide Mediterranean range, extending in the north to Central and Eastern Europe, and in the east to the Crimea, the Caucasus, and the Western Caspian region.

A list below summarizes a taxonomic composition of the *Lotus dorycnium* complex. We generally follow earlier studies regarding the limits of recognized taxa [1,9,11]. Their names are adjusted here to the present-day nomenclature and updated when necessary. The main reason for the taxonomic novelties proposed below is the need to accommodate the position of the group with the genus *Lotus*.

**Figure 1:**
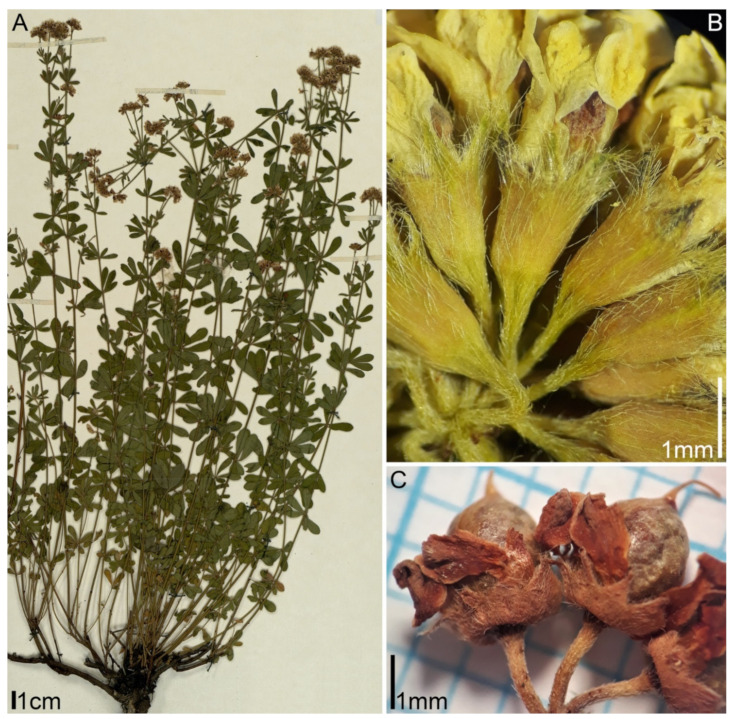
Morphology of *Lotus dorycnium* ssp. *herbaceus*: (**A**) habit; (**B**) flowers; (**C**) fruits. Herbarium specimens: (**A**) Crimea, D.D. Sokoloff s.n., MW0615046 (https://plant.depo.msu.ru/public/scan.jpg?pcode=MW0615046; accessed 16 January 2022); (**B**) Turkey, Tuzlacı 50735 (ISTE); (**C**) Turkey, S. Yüzbaşioğlu et al. 106380 (ISTE).

**Figure 2:**
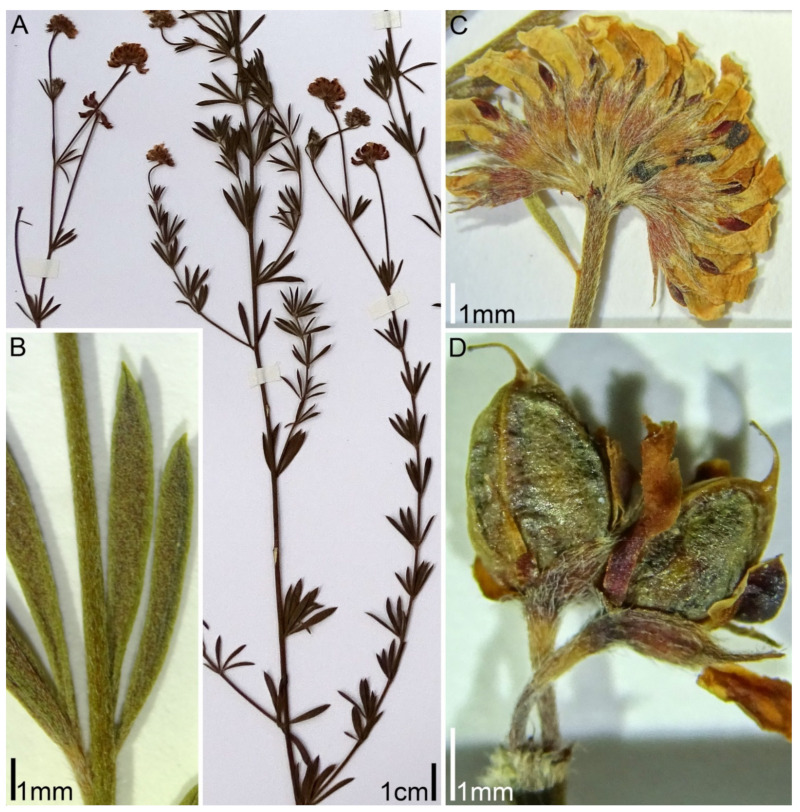
Morphology of *Lotus dorycnium* ssp. *gracilis*. Herbarium specimen GRAC1: Spain, S. Fos 50/05 (MA 774818): (**A**) flowering shoots; (**B**) detail of leaf; (**C**) umbel at anthesis; (**D**) fruits.

**Figure 3:**
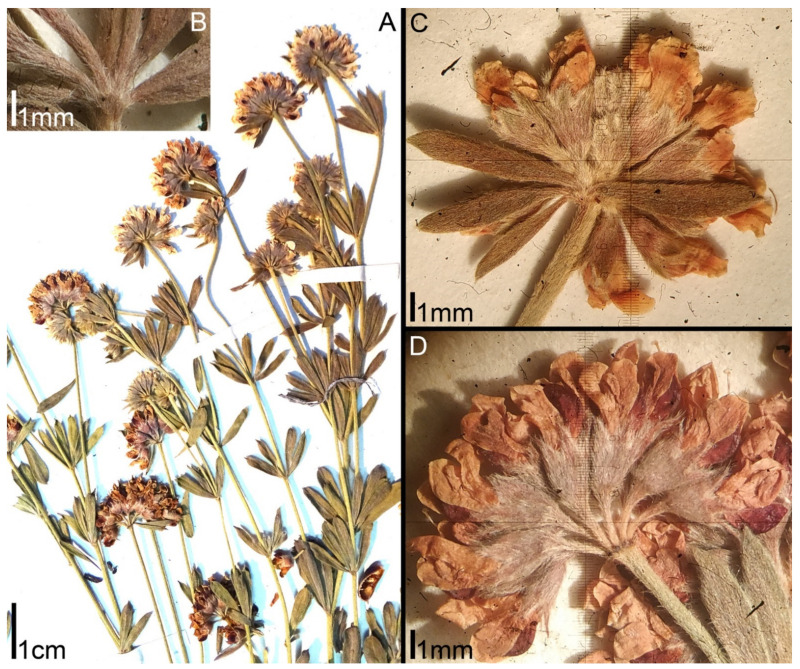
Morphology of *Lotus dorycnium* ssp. *germanicus*. Herbarium specimens: Slovakia, J.Ujcik 131 (LE) (**A**,**B**,**D**) and Hungary, Illarionova 13 (LE) (**C**): (**A**) fragments of flowering shoots; (**B**) leaf base; (**C**,**D**) umbels at anthesis.

**Figure 4:**
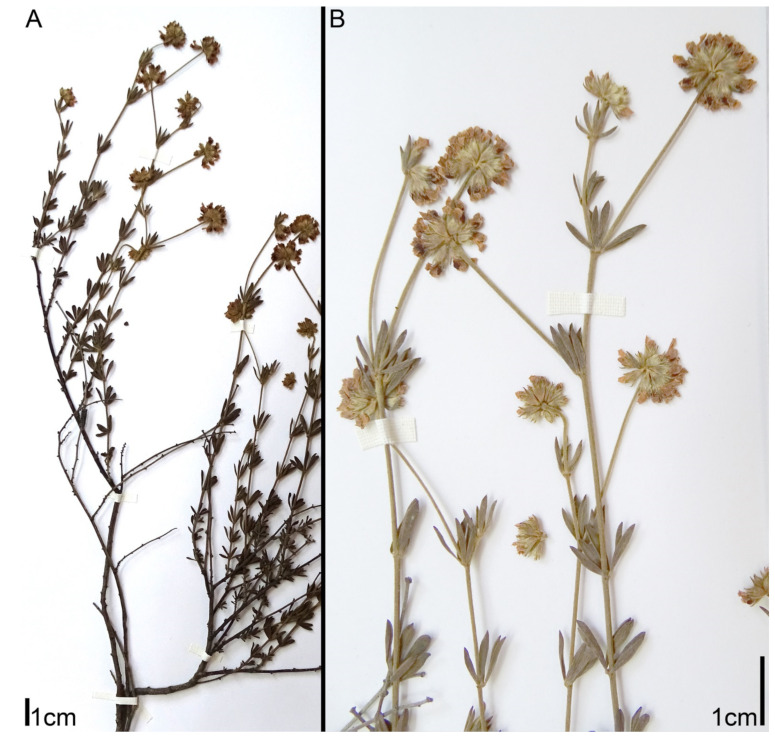
Morphology of *Lotus dorycnium* ssp. *dorycnium*. Herbarium specimen PENT03: Spain, E.Loriente s.n. (MA 658686): (**A**) habit; (**B**) fragments of flowering shoots.

**Figure 5:**
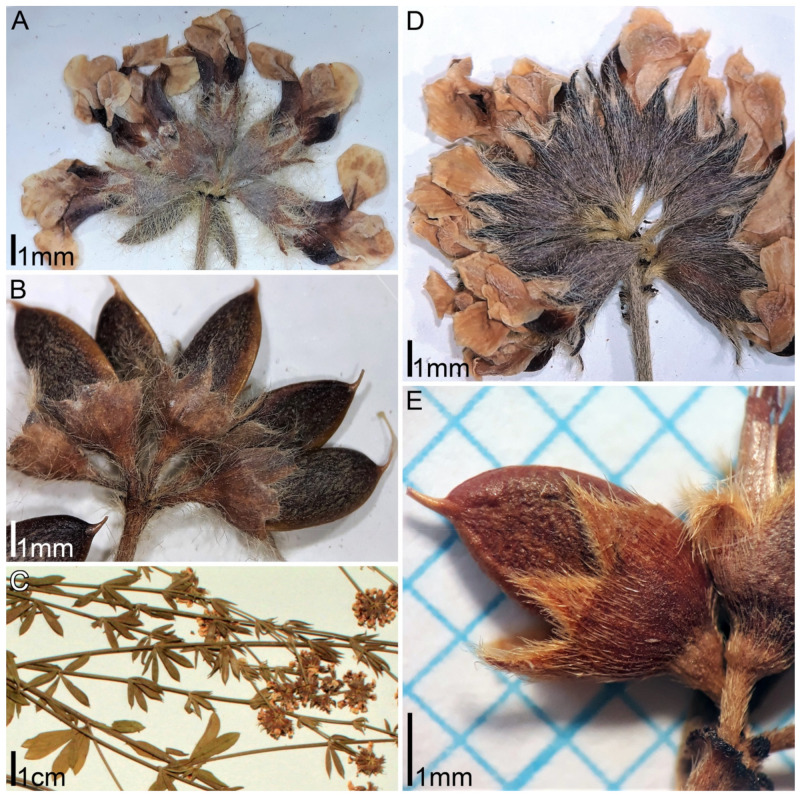
Morphology of *Lotus dorycnium* ssp. *anatolicus* (**A**,**B**) and *ssp. haussknechtii* (**C**–**E**): (**A**) umbel at anthesis (herbarium specimen: Turkey, M. Vural 967, GAZI); (**B**) fruits (herbarium specimen: Turkey, C. Birden 1421, GAZI); (**C**) fragments of flowering shoots (herbarium specimen: Turkey, H. Duman & F. Karavelioğulları 2232, GAZI); (**D**) umbel at anthesis (herbarium specimen: Turkey, A. Duran 2627, GAZI); (**E**) fruit (herbarium specimen: Turkey, Byfield & Pearman 73323, ISTE).

**Figure 6:**
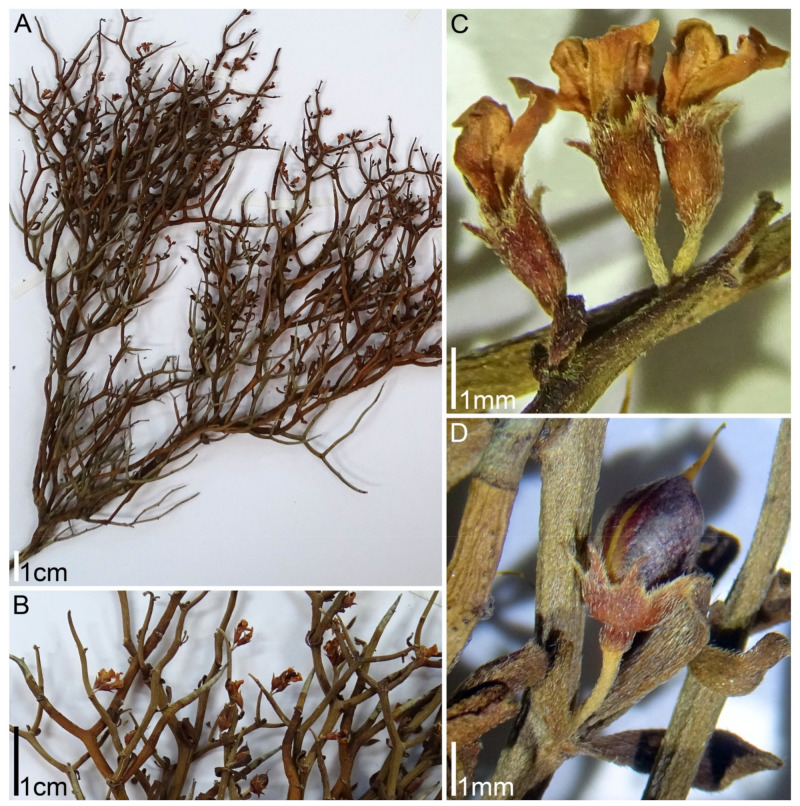
Morphology of *Lotus dorycnium* ssp. *fulgurans***.** Herbarium specimen: Spain, phare de Formentor, Majorque, A. Sotiaux 1 (MA 748212): (**A**) habit; (**B**) fragments of thorny shoots; (**C**) flowers; (**D**) fruit.

1. ***Lotus dorycnium* ssp. *herbaceus* (Vill.) Kramina & D.D. Sokoloff, comb. nov.** (Basionym: *Dorycnium herbaceum* Vill. 1779, Prosp. Hist. Pl. Dauphine: 44; Synonym: *Dorycnium intermedium* Ledeb.) (Figure 1). Distribution range: East Mediterranean, Balkan Peninsula, extending westwards to Italy and southeastern France, northwards to Germany, Poland, Czech Republic, Slovakia, and Transcarpathian Ukraine, eastwards to the Northern part of Asia Minor, the Crimea, the Caucasus, and Transcaucasia, and to the Western part of the Caspian region [11,12,13]. *Dorycnium* *intermedium* has been described from the Crimea as a species close to *D. herbaceum*, but differing from the latter mainly in a patent (not appressed) pubescence on the calyx [14]. Rikli [9] believed that *D. intermedium* and *D. herbaceum* do not differ either morphologically or geographically, but Steinberg [15] considered *D. intermedium* the easternmost race of *D. herbaceum*.
2. ***Lotus dorycnium* ssp. *gracilis* (Jord.) Kramina & D.D. Sokoloff, comb. nov.** (Basionym: *Dorycnium gracile* Jord. 1846, Obs. Pl. Crit. 3: 70; Synonyms: *Dorycnium herbaceum* ssp. *gracile* (Jord.) Nyman; *Dorycnium pentaphyllum* ssp. *gracile* (Jord.) Rouy; *Dorycnium jordanii* Loret & Barrandon; *Lotus jordanii* (Loret & Barrandon) Coulot, Rabaute & J.-M. Tison) (Figure 2). Distribution range: West Mediterranean: France, Spain, Balearic Islands, Algeria [9,11,13].
3. ***Lotus dorycnium* ssp. *germanicus* (Gremli) Kramina & D.D. Sokoloff, comb. nov.** (Basionym: *Dorycnium jordanii* subsp. *germanicum* Gremli 1889, Excursionsfl. Schweiz, Ed. 6.: 496; Synonyms: *Lotus germanicus* (Gremli) Peruzzi; *Dorycnium pentaphyllum* ssp. *germanicum* (Gremli) Gams; *Dorycnium germanicum* (Gremli) Rouy) (Figure 3). Distribution range: the largest continuous part: much of the former Yugoslavia, Albania, Northern Greece, W part of Bulgaria and southwestern Romania; minor part: Eastern Alps, Eastern Switzerland, Bavaria, and the Pannonian region [12].
4. ***Lotus dorycnium* ssp. *dorycnium*** (Synonyms: *Dorycnium pentaphyllum* ssp. *pentaphyllum; Dorycnium pentaphyllum* ssp. *suffruticosum* Bonnier & Layens; *Dorycnium pentaphyllum* ssp. *transmontaum* Franco) (Figure 4). Distribution range: Portugal and West Mediterranean (Spain, France, Italy, Algeria, Tunisia) [11,13].
5. ***Lotus dorycnium* ssp. *anatolicus* (Boiss. & Heldr.) Kramina & D.D. Sokoloff, comb. nov.** (Basionym: *Dorycnium anatolicum* Boiss. & Heldr. 1849, Diagn. Pl. Orient. ser. 1, 9: 31; Synonym: *Dorycnium pentaphyllum* ssp. *anatolicum* (Boiss. & Heldr.) Gams) (Figure 5A,B). Distribution range: Asiatic Turkey, Syria, Lebanon [11,13].
6. ***Lotus dorycnium* ssp. *haussknechtii* (Boiss.) Kramina & D.D. Sokoloff, comb. nov.** (Basionym: *Dorycnium haussknechtii* Boiss. 1872, Fl. Orient. 2: 163; Synonym: *Dorycnium pentaphyllum* ssp. *haussknechtii* (Boiss.) Gams) (Figure 5C–E). Distribution range: Asiatic Turkey, Syria, Lebanon, Bulgaria [11,13,16].
7. ***Lotus dorycnium* ssp. *fulgurans* (Porta) Kramina & D.D. Sokoloff, comb. nov.** (Basionym: *Anthyllis fulgurans* Porta 1887, Nuovo Giorn. Bot. Ital. 19: 303; Synonyms: *Dorycnium fulgurans* (Porta) Lassen; *Dorycnium pentaphyllum* subsp. *fulgurans* (Porta) Cardona, Lorens & Sierra) (Figure 6). Restricted to Balearic Islands. According to morphological [17,18] and molecular data [7], it is a member of the *L. dorycnium* complex; we have therefore included it in the study.

Diagnostic morphological characters of the taxa listed above are presented in Table 1. Greuter et al. [11] also included the Turkish endemics *Dorycnium amani* Zohary and *D. axilliflorum* Huber-Morath in their concept of the group of *D. pentaphyllum* (=*L. dorycnium* s.l.). *Dorycnium* *amani* was not included in the present study (or any other molecular-based work), because it is known from the type collection only and we had no material for this species. Besides, such a character of *D. amani* as a pronounced leaf rachis makes the inclusion of this species into the *L. dorycnium* complex debatable. The previous studies of *D. axilliflorum* demonstrated that it belongs to the *L.* *graecus* L. species group [7,8], rather than to the *L.* *dorycnium* complex.

**Figure 7:**
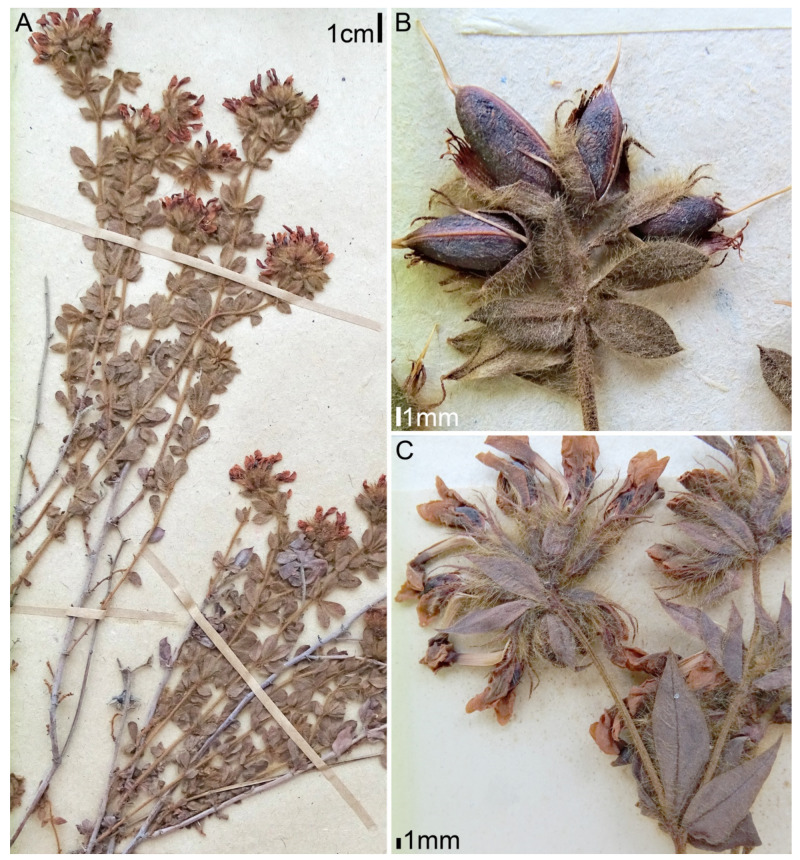
Morphology of *Lotus hirsutus*: (**A**) habit (herbarium specimen: Turkey, G. Ertem 25071, ISTE); (**B**) fruits (herbarium specimen: Turkey, A. Baytop et al. 10.063, ISTE); (**C**) umbels at anthesis (herbarium specimen: Turkey, A. & T. Baytop 7075, ISTE).

**Figure 8:**
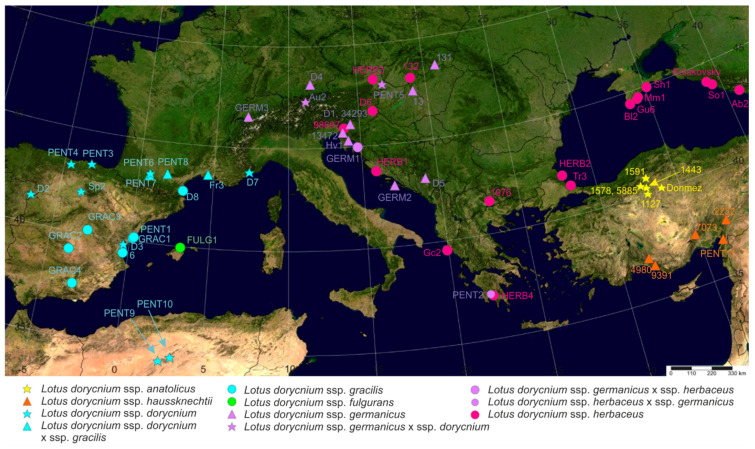
Geographical localities of specimens of the *Lotus dorycnium* complex studied here using molecular methods.

As the basic taxa recognized here within the *L. dorycnium* complex are subspecies, one should not expect that every single specimen can be identified up to that level. Indeed, some specimens included in the present study shared morphological and/or molecular features with two subspecies. For simplicity, these are tentatively called here hybrids, though in some instances more data should be accumulated to demonstrate clear evidence of reticulate evolution. Previous studies demonstrated that the *L. dorycnium* complex is not genetically isolated from *L. hirsutus* of *Lotus* sect. *Bonjeanea* (Figure 7) using plastid data, but monophyletic according to analyses of nuclear ribosomal markers ITS1-5.8S-ITS2 (nrITS) and 5′ETS, though sampling was relatively low [7,8,21]. Based on the nrITS data set, the *L. dorycnium* complex is younger than *L. strictus*, *L. rectus*, and *L. hirsutus* (estimated divergence times 2.52 Ma, 6.1 Ma, 4.94 Ma, and 4.16 Ma, respectively) [8]. Geographical distribution of the *Lotus dorycnium* complex and its subspecies based on specimens included in our molecular analyses is presented in Figure 8.

The main aims of the present study were: (1) to investigate genetic diversity and differentiation within the *L. dorycnium* complex across the whole distribution range using nrITS and a set of plastid DNA markers; (2) to compare degrees of genetic and morphological differentiation in this group; (3) to reveal geographic trends in genetic variability; (4) to refine data on interrelationships between the *L. dorycnium* complex and *L. hirsutus* using a more representative sampling of specimens and an enlarged set of plastid markers; (5) to test the hypothesis that nuclear markers allow precise separation of *L. hirstus* and the *L. dorycnium* complex.

## 2. Results

### 2.1. Morphometric Analyses

**Figure 9:**
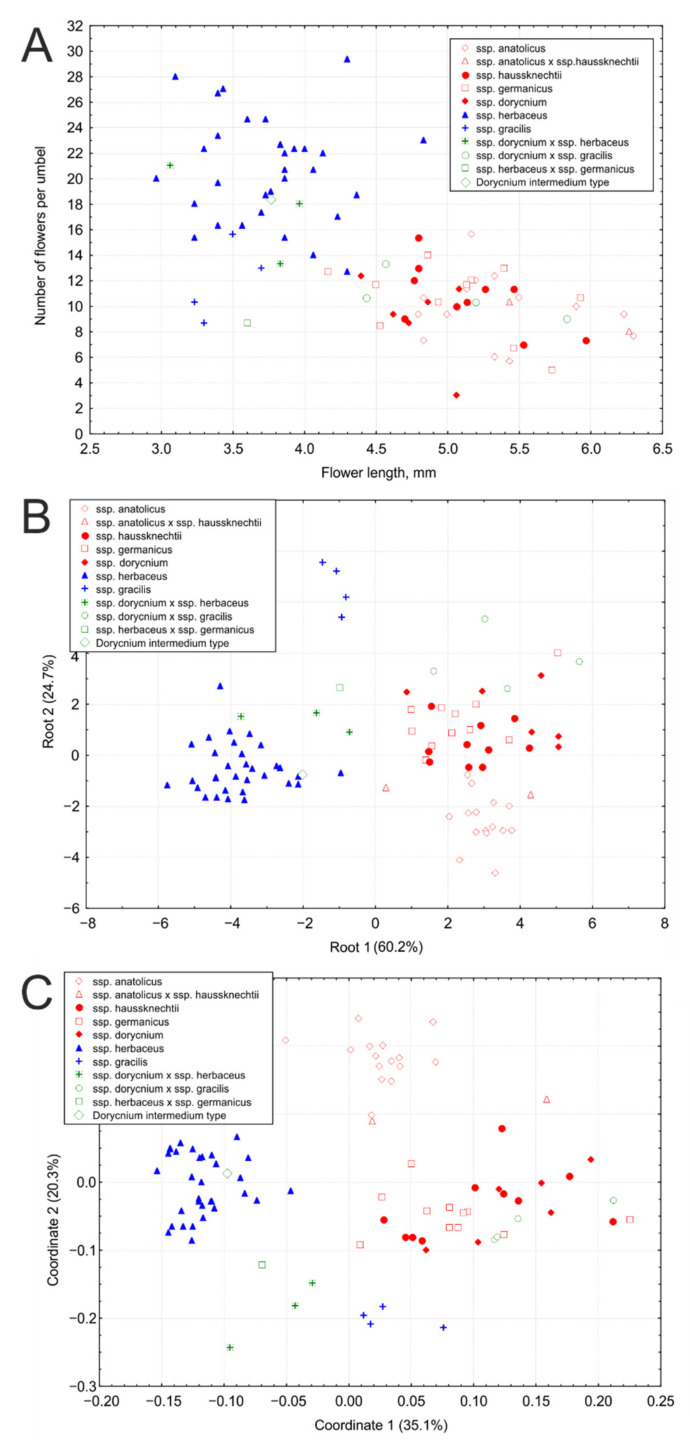
Results of morphometric analyses of the *Lotus dorycnium* complex. (**A**) Two-dimensional scatterplot of the specimens of the *L. dorycnium* complex by two morphological characters: OX—flower length (mm), OY—number of flowers per umbel; (**B**) Discriminant analysis of the specimens of the *L. dorycnium* complex; (**C**) Principal coordinate analysis of the specimens of the *L. dorycnium* complex.

Analysis of 89 individuals of the *L. dorycnium* complex using two characters (flower length and number of flowers per umbel) revealed a division of the dataset into two groups (Figure 9A):

1. Blue group: umbels with numerous (usually more than 13) small (usually shorter than 4.5 mm) flowers; this group includes ssp. *herbaceus* and ssp. *gracilis*.
2. Red group: umbels with generally less numerous, but larger flowers; this group includes ssp. *dorycnium*, ssp. *germanicus,* ssp. *anatolicus*, and. ssp. *haussknechtii*.

The hybrid specimens *L. d.* ssp. *dorycnium* × *L. d.* ssp. *herbaceus* were closer to ssp. *herbaceus* by these two characters, and *L. d. ssp. dorycnium* × *L. d.* ssp. *gracilis* on the opposite were closer to ssp. *dorycnium*. The hybrid specimen *L. d.* ssp. *herbaceus* × *L. d.* ssp. *germanicus* took the position between the two groups. The type specimen of *Dorycnium intermedium* included in the study for a comparison with other samples of *L. d*. ssp. *herbaceus* was placed in the center of the *L. d.* ssp. *herbaceus* cluster. The linear correlation coefficient between the variables “flower length” and “number of flowers per umbel” is −0.685 (*p* = 1.3204 × 10^−12^).

In DA, we set the group numbers for ‘pure’ subspecies only: ssp. *anatolicus*, ssp. *haussknechtii,* ssp. *dorycnium,* ssp. *germanicus,* ssp. *herbaceus*, and ssp. *gracilis*. The specimens of presumed hybrid origin were left without a group number. The analysis revealed three main clusters in the dataset (Figure 9B): 1. ssp. *herbaceus;* 2. ssp. *gracilis;* 3. ssp. *dorycnium*, ssp. *germanicus,* ssp. *anatolicus*, and ssp. *haussknechtii.* In the third group, ssp. *anatolicus* differs slightly from others along the second axis. The specimens *L. d.* ssp. *anatolicus* × *L. d.* ssp. *haussknechtii* took the position between corresponding subspecies. The positions of other hybrid specimens and the type specimen of *D. intermedium* were similar to those obtained on a 2D scatterplot. Note that we were unable to sample type material of *Dorycnium herbaceum* or any other specimen from the region from which it was described (southeastern France).

PCoA analysis using 24 morphological characters revealed four main clusters (Figure 9C): 1. ssp. *herbaceus;* 2. ssp. *gracilis;* 3. ssp. *dorycnium*, ssp. *germanicus*, and ssp. *haussknechtii*; 4. ssp. *anatolicus.* The position of hybrid specimens was similar to previous analyses, but the specimens *L. d.* ssp. *dorycnium* × *L. herbaceus* and *L. herbaceus* × *L. germanicus* were located between the Clusters 1 and 2.

All three methods of analysis demonstrated the absence of clear morphological differentiation within the group that includes *L. dorycnium* ssp. *dorycnium*, *L. d.* ssp. *haussknechtii*, and *L. d.* ssp. *germanicus*.

### 2.2. Phylogenetic Analysis of nrDNA ITS1-2 Dataset

The nrITS dataset included 125 accessions (Supplementary Materials, Dataset S1), 64 accessions from the *Lotus dorycnium* complex, 58 from other *Lotus* species, and three accessions from outgroups represented by *Hammatolobium kremerianum*, *Cytisopsis pseudocytisus*, and *Tripodion tetraphyllum*. For each of the samples L7 of *L. corniculatus*, 425 of *L. cytisoides*, and GRAC2 of *L. dorycnium* ssp. *gracilis* (Table 2), two different nrITS sequences were obtained through direct sequencing (without cloning). The total alignment length was 672 bp (601 bp after the exclusion of gap-rich and ambiguous positions). From 601 sites, 252 were variable and 175 parsimony informative.

**Figure 10:**
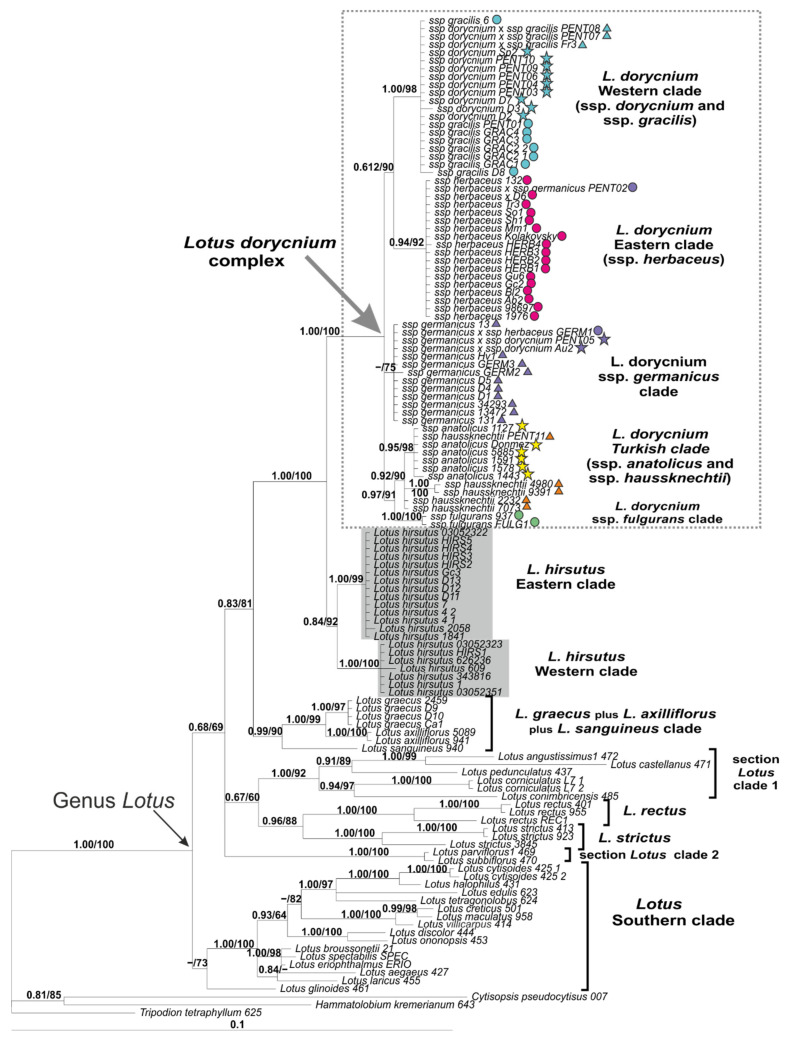
Phylogenetic relationships in *Lotus* with expanded representation of the *L. dorycnium* complex inferred from Bayesian analysis of the nrITS dataset. Branch lengths are proportional to the number of expected nucleotide substitutions, scale bar corresponds to 0.1 substitutions per site. Numbers above branches are posterior probabilities. Numbers below branches or after slashes are bootstrap support values found in maximum likelihood (ML) analysis of the same dataset (values equal to or more than 0.6/60% shown). See Table 2 and Appendix A for voucher information.

The Bayesian phylogenetic tree topology is presented in Figure 10 where posterior probabilities (PP) of nods are given together with bootstrap support (BS) obtained in a maximum likelihood (ML) analysis constructed using IQ-tree software. The results obtained in the ML analysis conducted using RAxML software generally correlate with those obtained in IQ-tree. ML trees based on the nrITS dataset are presented in Supplementary Materials (Figures S1 and S3). Both the Bayesian and ML analyses revealed a highly supported monophyly of the genus *Lotus*. All specimens of the *Lotus dorycnium* complex were clustered within a highly supported clade (PP = 1.00/BS = 100%), which was sister to a less supported *L. hirsutus* clade. The latter was subdivided into Eastern and Western subclades. *Lotus graecus* and related species (*L. axilliflorus* and *L. sanguineus*) were more distantly related to the clade of *L. dorycnium* and *L. hirsutus*.

Within the *Lotus dorycnium* complex clade, the following subclades can be distinguished, listed below in descending order of support: (1) *L. d.* ssp. *fulgurans* clade (1.00/100%). (2) *L. dorycnium* Western clade (1.00/98%), represented by an unresolved mixture of *L. d.* ssp. *dorycnium*, ssp. *gracilis* and their hybrids occurring in the western part of the area, i.e., in Spain, France, Portugal, and Algeria. (3) *L. dorycnium* ssp. *herbaceus* clade (0.94/92%). (4) The clade *of L. d.* ssp. *anatolicus* and ssp. *haussknechtii* represented by Turkish specimens (0.92/90%). (5) *L. d.* ssp. *germanicus* clade, not supported by both methods of analysis (0.54/75%). The ITS analysis moderately supports sister relationships between *L. d.* ssp. *fulgurans* and a clade that combines ssp. *anatolicus* and ssp. *haussknechtii*, which contradicts the geographical distribution of these taxa.

### 2.3. Phylogenetic Analysis of the Plastid DNA Dataset

The concatenated plastid dataset included 122 accessions (Supplementary Materials, Dataset S2) of the same composition as nrITS dataset. The total alignment length of the plastid DNA dataset was 2361 bp (including *trn*L-F 967 bp, *rps*16 intron 914 bp, and *psb*A-*trn*H 480 bp). After the exclusion of gap-rich and ambiguous positions, the alignment length was reduced to 2047 bp, from which 354 sites were variable and 187 parsimony informative.

**Figure 11:**
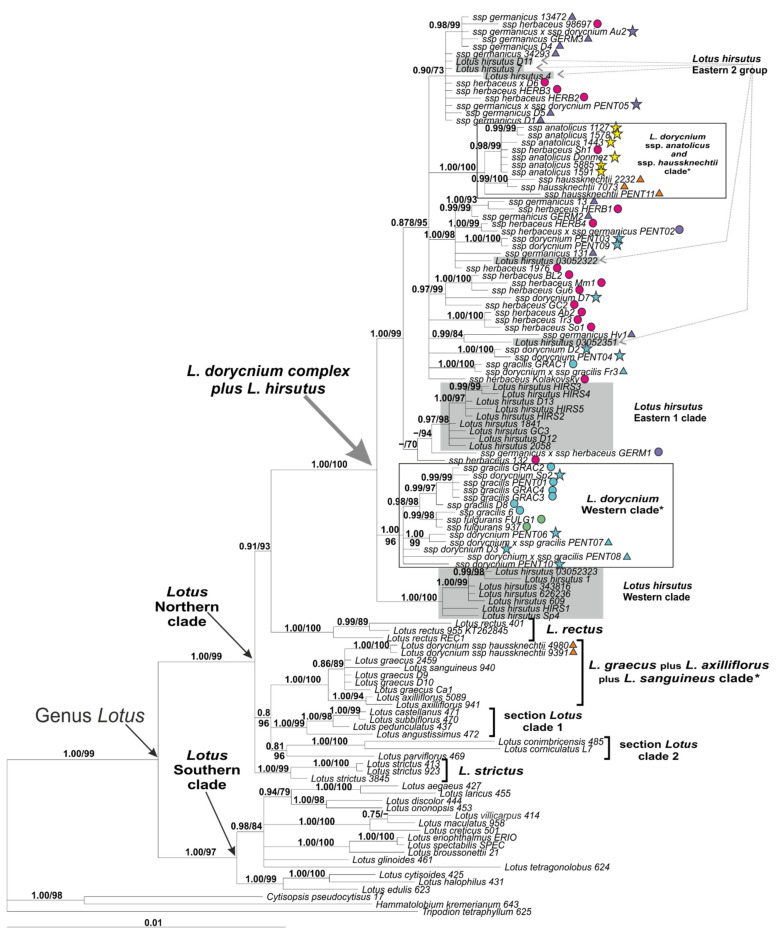
Phylogenetic relationships in *Lotus* with expanded representation of the *L. dorycnium* complex inferred from Bayesian analysis of the plastid DNA dataset. Branch lengths are proportional to the number of expected nucleotide substitutions; scale bar corresponds to 0.01 substitutions per site. Numbers above branches are posterior probabilities. Numbers below branches or after slashes are bootstrap support values found in ML analysis of the same dataset (values equal to or more than 0.6/60% shown). Clades slightly differing in composition from the corresponding clades in the ITS tree are marked with an asterisk. See Table 2 and Appendix A for voucher information.

The Bayesian phylogenetic tree constructed by the plastid DNA dataset is presented in Figure 11 and supplied by PP values and BS values of nods obtained in ML analysis conducted using IQ-tree, as in the analysis of the ITS dataset. ML trees based on the plastid dataset are presented in Supplementary Materials (Figures S2 and S4). The monophyly of the genus *Lotus* was supported by both Bayesian and ML methods (PP = 1.00/BS = 99%). Two main clades within *Lotus*, namely *Lotus* Northern clade and *Lotus* Southern clade, were well confirmed (1.00/99% and 1.00/97%, respectively). All samples of the *Lotus dorycnium* complex were placed in the *Lotus* Northern Clade; however, they did not form a separate subclade, but were combined with *L. hirsutus* within a highly supported common clade (1.00/100%). Within this common clade, examined specimens of *L. hirsutus* formed two subclades (Western subclade and Eastern 1 subclade) and several specimens were scattered among the specimens of the *L. dorycnium* complex (Eastern 2 group). None of the clades within the *L. dorycnium* complex revealed in ITS analyses were observed in analyses based on plastid markers. Only two subclades more or less corresponding to the clades in the ITS analyses were found. These were (1) a clade combining accessions of the ssp. *anatolicus* and ssp. *haussknechtii* and (2) the *L. dorycnium* Western clade, but their composition differed slightly from those obtained by ITS data. In accordance with its distribution in the Balearic Islands, *Lotus d.* ssp. *fulgurans* was revealed as a member of the *L. dorycnium* Western clade. The most surprising result from the analysis of the plastid DNA is the very well supported placement of two samples of *L. d.* ssp. *haussknechtii* (4980 and 9391) within the clade that includes *L. graecus* plus related taxa.

### 2.4. Haplotype Network Based on the Concatenated Plastid DNA Dataset

**Figure 12:**
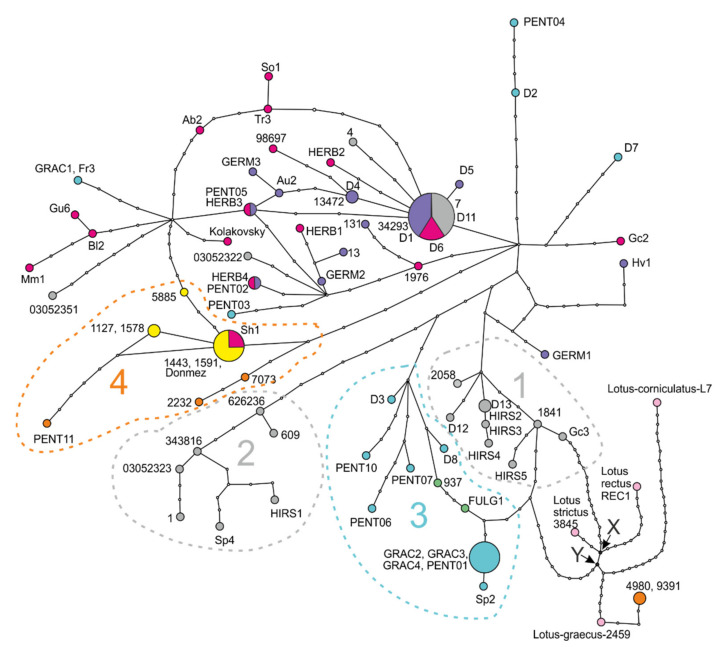
Plastid DNA haplotype network reconstructed from a combined plastid DNA dataset. The size of each circle is proportional to the frequency of the haplotype in the dataset. The haplotype colors correspond to the colors in Figure 1, Figure 3, and Figure 4. *Lotus hirsutus* is gray. The outgroups, represented by *L. strictus*, *L. rectus*, *L. graecus*, and *L. corniculatus*, are pink. Groups of haplotypes marked with dashed lines: 1, Eastern group of *L. hirsutus*; 2, Western group of *L. hirsutus*; 3, Western group of *L. dorycnium*; 4, Turkish group of *L. dorycnium*.

TCS analysis included 85 sequences of concatenated plastid DNA regions *trn*L-F, *rps*16 intron, and *psb*A-*trn*H, 60 sequences of the *L. dorycnium* complex, 21 sequences of *L. hirsutus*, and four sequences of the outgroup (*L. corniculatus*, *L. rectus*, *L. strictus*, *L. graecus*). Sixty-seven haplotypes have been revealed in the dataset, four in the outgroup, and 63 in *L. hirsutus* and *L. dorycnium* s.l. (Figure 12). The majority of the haplotypes, 58 of 67 (or 86,6%), are singletons. We revealed three haplotypes shared between subspecies of *L. dorycnium* and one haplotype shared between *L. dorycnium* and *L. hirsutus*. Three of the shared haplotypes were inner, and only one haplotype, shared between HERB4 and PENT02 specimens, was a tip haplotype.

To study genetic diversity within the *L. dorycnium* complex and *L. hirsutus*, we divided all specimens of these taxa according to their geographical origin (Figure 8, [8]) and placement in the clades of the ITS phylogenetic tree (Figure 10). Thus, we have three geographical groups of specimens of *L. dorycnium* s.l. (Western, Eastern and Turkish) and two geographical groups of specimens of *L. hirsutus* (Western and Eastern). We found fairly high levels of genetic diversity in these geographical groups (Table 3).

Within the *Lotus dorycnium* complex, maximal haplotype diversity was observed in the eastern group (0.986), but its nucleotide diversity is of middle value (0.0043). The western group, on the contrary, had maximal nucleotide diversity among the studied populations (0.0057), and middle Hd. The Turkish geographical group is characterized by minimal population diversity parameters, both Hd and Pi (Table 3). The eastern group demonstrated a unimodal mismatch distribution, suggesting recent population expansion [22], while the other groups of the *L. dorycnium* complex showed multimodal distribution patterns indicating a stable population size over time.

Both geographical groups of *L. hirsutus* were characterized by high levels of haplotype diversity and average values of nucleotide diversity, which together with the multimodal nature of mismatch distribution suggests a long existence in the given territory. At the same time, the western group of the species has higher indicators of diversity than the eastern one.

The haplotype network does not allow for determining an exact ancestral haplotype of the *Lotus dorycnium* complex, because of many loops occurring in the network. The hypothetical haplotypes X and Y may be the divergence points between the studied clade (i.e., the *L. dorycnium* complex and *L. hirsutus*) and outgroups. The haplotype group #1 (*L. hirsutus* Eastern 1 clade) and group #3 (*Lotus dorycnium* Western clade) are the closest to the hypothetical haplotypes X and Y. Two *L. hirsutus* haplotype groups (Eastern group (#1) and Western group (#2)) and two *L. dorycnium* complex haplotype groups (Western group (#3) and Turkish group (#4)) have the most pronounced branching character without loops or with single loops. Other haplotypes, mainly representing *L. dorycnium* ssp. *herbaceus*, *L. dorycnium* ssp. *germanicus*, and *L. hirsutus* specimens, form a very complex and difficult-to-interpret part of the network with several loops and three out of four shared haplotypes.

The networks constructed separately for each plastid DNA marker (not presented), despite differences in details, demonstrated the following common features: the presence of loops, many missing/hypothetical haplotypes (differences between haplotypes with two or more mutations), the presence of a haplotype shared by *L. d.* ssp. *germanicus*, *L. d.* ssp. *herbaceus* and *L. hirsutus*.

## 3. Discussion

### 3.1. Taxonomic Identification of Specimens

For the taxonomic identification of the specimens, we used keys from several sources [1,9,15,19,20]; however, only the account of Rikli [9] covers the studied group worldwide, though it provides rather short keys and does not consider a lot of observations and collections made during last 120 years. Note that Rikli did not include in his account the peculiar Balearic endemic known at that time as *Anthyllis fulgurans* [23] and only much later identified as a member of *Dorycnium* [24]. The rest of the works used here for identification of samples are of a regional scale and do not include all taxa of the complex, which makes it difficult to compare taxa from different regions. The use of fruit characters was restricted because the majority of the specimens studied were in the flowering phase. Another problem was the ambiguity in the formulation of some quantitative features. For example, when the authors use the calyx tube length [1,9,19,20], they do not specify whether the tube length includes a hypanthium. In a few cases, for taxonomic identification, we considered the geographical location and phylogenetic position of a specimen.

The results of our morphometric analyses demonstrated that the *Lotus dorycnium* complex can be subdivided into two groups by flower length and the number of flowers per umbel, which agrees with the ideas of Rikli [9]. These characters make it possible to separate ssp. *herbaceus* and ssp. *gracilis* with small flowers and multi-flowered inflorescences from the rest of the complex. Between themselves, these two subspecies (ssp. *herbaceus* and ssp. *gracilis*) differ in the length of the calyx teeth and pubescence. The use of additional traits, especially those of pubescence, distinguishes ssp. *anatolicus* from other representatives of the group with large flowers and few-flowered inflorescences. However, even the use of a large number of morphological characters does not allow for precise separation of ssp. *dorycnium*, ssp. *germanicus*, and ssp. *haussknechtii* from each other. In addition, we found ten samples of intermediate morphology between subspecies or even between two groups of subspecies, considered here as intersubspecific hybrids. Ball [1] noted the difficulty of separating taxa within the *L. dorycnium* complex on a purely morphological basis and treated them as subspecies of *Dorycnium pentaphyllum* Scop. Demiriz [19] also accepted the subspecific rank of taxa within *D. pentaphyllum* Scop. Hybridization between the members of the *Lotus dorycnium* complex is well known [12,17,18]. An introgression zone between ssp. *herbaceus* and ssp. *germanicus* in southern Moravia and western Slovakia has been described [12]. Hybrids between ssp. *dorycnium* and ssp. *fulgurans* have been discovered in the island of Minorca among the Balearic Islands, and their hybrid nature has been confirmed by molecular methods [17,18]. The available evidence suggests the subspecies rank of the studied taxa of the *L. dorycnium* complex.

### 3.2. Phylogenetic Placement of Lotus dorycnium s.l. and Relationships among Its Subspecies

The phylogenetic analyses conducted using nrITS and plastid datasets support earlier results on the phylogenetic position of the *Lotus dorycnium* complex within the genus *Lotus* [7,8]. The ITS data confirmed sister relationships between the *L. dorycnium* complex and *L. hirsutus*.

The structure of the clade of the *Lotus dorycnium* complex in the nrITS phylogenetic trees reflects its geographical differentiation. Three of four large subclades (i.e., the Western, Eastern, and Turkish ones) represent three geographical groups of specimens. On the other hand, specimens of ssp. *germanicus*, distributed in an area that partially overlaps with that of ssp. *herbaceus* do not form a statistically supported clade. Strong or weak segregation of eastern and western evolutionary lineages within widely distributed Mediterranean species has been described for many groups of plants and animals [25,26]. However, clustering of ssp. *fulgurans* from the western Mediterranean (Balearic Islands) with the Turkish clade is inconsistent with geography. Unfortunately, our study did not include any samples of the *L. dorycnium* complex from the middle parts of the Mediterranean region, such as the Italian Peninsula and the large islands Corsica, Sardinia, and Sicily. Further investigation of the material from these regions may shed light on the evolutionary history of ssp. *fulgurans*. Interestingly, ssp. *fulgurans* resembles in habit another local endemic taxon of the Balearic Islands that also belongs to the tribe Loteae, *Anthyllis hystrix* (Willk. ex Barceló) Cardona, Contandr. et Sierra. Both plants are thorny shrublets (ssp. *fulgurans* is the only thorny member of *Lotus*). *Anthyllis hystrix* appears to share some aspects of its phylogeography with *L. d.* ssp. *fulgurans*. Like *L. d.* ssp. *fulgurans*, *A. hystrix* has all its potential relatives occurring to the east of its range. Plastid and nuclear data revealed a remarkable incongruence regarding the position of *A. hystrix* [27], and this result, together with the octoploid chromosome number, supports its possible hybrid origin [28]. However, the chromosome number of *L. d.* ssp. *fulgurans*, is the same as in other members of the *L. dorycnium* complex, 2*n* = 14 [29].

Within the highly supported Western subclade of the *L. dorycnium* complex, the specimens of ssp. *pentaphyllum* and ssp. *gracilis* are not separated, which implies the presence of a gene flow between the two subspecies. The same can be addressed to a pair of taxa from Turkey, ssp. *anatolicus* and ssp. *haussknechtii*.

The phylogenetic analyses by plastid DNA dataset demonstrated a clear separation of a clade consisting of the *L. dorycnium* complex and *L. hirsutus* in the phylogenetic trees. At the same time, they showed the impossibility of separating these two taxa based on plastid data, which confirms the results obtained earlier using a more restricted material [7,8,21]. As a whole, plastid markers are of limited value for defining taxonomic boundaries within the studied complex. Moreover, the geographic structure within the *L. dorycnium* complex revealed by nrITS is much less pronounced in the analysis of plastid DNA.

### 3.3. Phylogeography of the Lotus Dorycnium Complex

Many loops present in the plastid haplotype network suggest homoplastic mutations that are common in plastid sequences [30,31]. These loops and the general network pattern do not imply a single ancestral haplotype of the *Lotus dorycnium* complex. It is possible that the origin of the complex is associated with a number of hybridization events. *Lotus hirsutus* is obviously the closest species to the *L. dorycnium* complex. Their relationships are not completely resolved in analyses of plastid data, but rather are resolved in ITS analyses. This can be explained by the lower evolutionary rate of plastid sequences, which, given the recent evolution of the group [8], may not be sufficient to differentiate genetic lines. In contrast, the concert evolution of nrITS may play an important role in the evolution of low-level taxa, acting as a process analogous to lineage sorting [31]. Most of the shared haplotypes are internal, and the derived haplotypes are subspecies-specific, suggesting retention of ancestral variability and incomplete lineage sorting, e.g., [32,33] rather than recent hybridization. However, it is not so easy to distinguish between these two processes.

The haplotype network of the *L. dorycnium* complex is branched, with many missing/hypothetical haplotypes and a predominance of singletons. The network of another complex of species from the genus *Lotus*, the *L. corniculatus* complex, contains several widespread haplotypes, each of which has several derived haplotypes, mainly differing in one mutation [34]. Despite the differences in methodology (the *trn*L-F plastid DNA region in *L. corniculatus* and three plastid DNA regions in *L. dorycnium*), it is possible to outline several important differences between these networks, associated with the different histories of these complexes. It is assumed that the origin of both complexes is associated with the Mediterranean, and then the members of the complexes spread to more northern and eastern regions. Some representatives of the *L. corniculatus* complex (for example, *L. krylovii* Schischk. & Serg.) have migrated much further to the north and east and have undergone a recent expansion there, as evidenced by the presence of widespread haplotypes and a low number of derived haplotypes. In contrast, most subspecies of *L. dorycnium* apparently existed for a long time in the Mediterranean region, undergoing fluctuations in abundance, as evidenced by the presence of many missing haplotypes and the multimodal distribution of pairwise substitutions. *L. d.* ssp. *herbaceus* is the subspecies, the most advanced to the east. We hypothesize that it may have undergone a relatively recent expansion, as evidenced by the unimodal mismatch distribution.

Remarkably, the Western clade of *L. hirsutus* and the Western clade of *L. dorycnium* are both well-supported in the plastid phylogeny, whereas eastern accessions of both species are intermixed with each other. Nuclear data, again, show a well-supported western clade in *L. dorycnium* (though it does not include ssp. *fulgurans*). These data indicate that the evolutionary processes responsible for data incongruence between plastid and nuclear sequences of these two morphologically well-defined species were most likely localized in the eastern part of the Mediterranean region.

In the eastern Mediterranean, in southern Turkey, two other members of the *L. dorycnium* complex with incongruent position in phylogenetic trees constructed using plastid and nuclear data were discovered in this study. These two samples, 4980 and 9391, were identified as *L. dorycnium* ssp. *haussknechtii*, which agrees with their clusterization within the *L. dorycnium* Turkish clade in the ITS trees. However, according to plastid data, these specimens turned out to be close to *L. graecus* L., a species not included in the *L. dorycnium* complex. This result suggests gene exchange between the two taxa occurring in recent or older times.

## 4. Materials and Methods

### 4.1. Plant Material

The molecular study involved 122 specimens, including 61 specimens of *Lotus dorycnium* complex, 21 specimens of *L. hirsutus*, 37 specimens of other *Lotus* species representing all main sections of the genus, and 3 specimens of genera *Cytisopsis*, *Hammatolobium*, and *Tripodion*, closely related to *Lotus*. Samples for molecular studies were taken from herbarium specimens stored in herbaria ANK, GAZI, LE, MA, MHA, MW, P, and ZA. Voucher information and GenBank accession numbers are presented in Table 2 and Appendix A. Geographical distribution of specimens included in molecular analyses is presented on a map (Figure 8) prepared using SimpleMappr [35].

The morphological study was conducted on herbarium specimens and involved 89 specimens belonging to the *Lotus dorycnium* complex stored in herbaria GAZI, ISTE, LE, MA, MHA, MW, P, and ZA. Voucher information of specimens included in the morphological analysis only is presented in Appendix B.

### 4.2. DNA Extraction, Amplification, and Sequencing

DNA was extracted from herbarium specimens (ca. 20 mg of leaf tissue) with NucleoSpin Plant II kit (Macherey-Nagel, Germany) according to the manufacturer’s instructions or using the CTAB method [36]. The nrDNA ITS and plastid DNA *psb*A-*trn*H intergenic spacer, *trn*L-*trn*F intergenic spacer and *trn*L intron, and *rps*16 intron were selected for the analysis. The sequences of the nrITS were amplified with primers NNC-18S10, C26A [37], ITS2, and ITS3 [38]. The amplification of the *psb*A-*trn*H spacer was conducted using primers trnH2 [39] and psbAF [40]. The sequences of the *trn*L-*trn*F region of plastid DNA were amplified using standard primers ‘c’, ‘d’, ‘e’ and ‘f’ [41], and the sequences of *rps*16 intron using primers rpsF, rpsR2 [42], Lot-rps16-F and Lot-rps16-intR [8]. PCRs were performed in a 0.02 mL mixture containing 10–20 ng DNA, 3.2 pmol of each primer and MasDDTaqMIX (Dialat LTD, Moscow, Russia) containing 0.2 mM of each dNTP, 1.5 mM MgCl2, and 1.5 units of SmarTaqDNA polymerase. Amplification of nrITS region and all plastid DNA regions was performed under the following conditions: hold 94 °C, 3 min; 94 °C, 30 s; 57 °C, 40 s; 72 °C, 60 s; repeat 30 cycles; extend 72 °C, 3 min. 

PCR products were purified using the Cleanup Mini kit (Evrogen, Moscow, Russia) and then used as a template in sequencing reactions with the ABI Prism BigDye Terminator Cycle Sequencing Ready Reaction Kit v. 3.1. Sequencing was performed on the ABI PRISM 3100 genetic analyzer (Applied Biosystems, Foster City, CA, USA). Forward and reverse strands of all samples were sequenced. The polymorphism of ITS within one specimen was detected by direct sequencing (without cloning), by the presence of double peaks on an electropherogram.

The sequences were aligned using MAFFT version 7.215 [43,44] and then adjusted manually in BioEdit version. 7.2.5 [45]. The matrices of *psb*A-*trn*H spacer, *rps*16 intron, and *trn*L-F plastid DNA regions were combined into a single matrix. Gap-rich and ambiguous positions were excluded from the analyses. The aligned data matrices are presented in the online Supplement (Datasets S1–S2).

### 4.3. Phylogenetic Analyses

Maximum likelihood analyses were performed in IQ-tree version 2.1.1 [46], internal branch support was assessed using the ultrafast bootstrap [47] with 10,000 re-samplings. The GTR + R model of nucleotide substitutions for plastid data and the SYM+ Γ model for nrITS were selected as the most appropriate by the Bayesian information criterion in the built-in ModelFinder utility [48]. In addition, ML analysis with 500 nonparametric bootstrap re-samplings was performed and a majority rule consensus tree was constructed for both data sets in RAxML version 8.2.10. The GTR + G model was used and each tree search procedure started with a random tree [49].

The Bayesian inference was performed using MrBayes v. 3.2.6 [50] considering the optimal model of nucleotide substitutions selected by AICc in PAUP version 4.0a [51] for each marker: SYM + Γ (symmetrical model with substitution rate heterogeneity) for nrITS, and GTR + Γ for plastid data. The Bayesian analysis used four independent runs of 25 million generations and four chains sampling every 1000th generation. Non-convergence assessment and burn-in estimation was carried out in VMCMC ver. 1.0.1 [52]. The first two million generations were discarded as burn-in and the remaining trees from both runs were combined in a 50% majority-rule consensus tree.

Phylogenetic relationships among the plastid DNA haplotypes were reconstructed using statistical parsimony analysis as implemented in TCS v1.2 [53]. Long indels were reduced to one character, then gaps were treated as fifth state. *L. rectus*, *L. strictus*, *L. hirsutus*, *L. graecus*, and *L. corniculatus* were used as outgroups. At the first stage, the haplotype networks were constructed separately for each plastid DNA marker (not presented), then for the concatenated set of three markers (*trn*L-F, *rps*16 intron, and *psb*A-*trn*H). Parameters of genetic variability were calculated using DnaSP 6 software [54].

### 4.4. Morphometric Analyses

The following 28 morphological characters were studied: 1. Stem length (cm); 2–4. Upper, lateral, and lower calyx teeth width (mm); 5. Average calyx teeth width (mm); 6–8. Upper, lateral, and lower calyx teeth length (mm); 9. Average calyx teeth length (mm); 10. Calyx tube length (mm); 11. Calyx tube length (including hypanthium) (mm); 12. Flower length (mm); 13. Pedicel length (mm); 14. Number of flowers per umbel; 15. Basal leaflet length (mm); 16–18. Length (mm), width (mm) and index (length to width ratio) of a terminal leaflet of a middle stem leaf; 19–21. Length (mm), width (mm), and index (length-to-width ratio) of a terminal leaflet of an upper stem leaf; 22. Index 1 (calyx tube length to calyx teeth length ratio); 23. Index 2 (pedicel length to calyx tube length ratio); 24–26. Average trichome length on stems, leaves and calyces (mm); 27–28. Degree of pubescence deviation on stems and leaves (ranks) (1—only appressed hairs; 2—appressed and patent hairs; 3—only patent hairs). Three measurements of each trait were carried out on the plant, and then the data were averaged. 

To test the hypothesis about subdivision of the *L. dorycnium* complex into two main groups according to flower length and number of flowers in a head [9], we constructed a 2D scatterplot with these two characters. Then, to test the hypothesis about the separation of taxa within the *L. dorycnium* complex by a set of quantitative morphological characters, we conducted a discriminant analysis (DA) using Statistica v.7.0 for Windows [55]. Finally, to test the hypothesis about the separation of taxa within the studied complex by a set of quantitative and qualitative characters, we performed a principal coordinate analysis (PCoA) using PaSt 4.08 software [56]. For the PCoA method we used the Gower distance metric, which is suitable for a combination of quantitative and qualitative characters.

## 5. Conclusions

Our phylogenetic and phylogeographic study of the *Lotus dorycnium* L. (=*Dorycnium pentaphyllum* Scop.) complex revealed a tendency towards a geographical differentiation into Western, Eastern (more precisely, north-eastern) and Turkish groups supported by nrITS data. The analysis of the same set of specimens using plastid markers demonstrated a low resolution both between the *L. dorycnium* complex and *L. hirsutus* and among the taxa of the *L. dorycnium* complex. Interestingly, our plastid phylogeny also revealed some geographical differentiation, but in a different way. Namely, our plastid tree has a well-supported clade that combines accessions of *L. dorycnium* s.l. and *L. hirsutus* from the eastern parts of their ranges (including Turkey), whereas western accessions of the two species form separate clades.

The discordance between our plastid and nuclear data can be interpreted as evidence of an incomplete lineage sorting and/or hybridization. The only potential way of further improving plastid phylogenetic data is though the generation of numerous complete plastid genomes to see whether current features of plastid phylogeny can be partly explained by the still inadequate informativeness of the markers used so far. However, experience from other plant groups suggests that phylogenies inferred from complete plastid genomes may still be incongruent with morphology and taxonomy. A recent study of *Ophrys* (Orchidaceae) in Europe and the Mediterranean revealed that plastomes represent geographic location more strongly than taxonomic assignment and correlate poorly with morphology, suggesting widespread plastid capture and possibly post-glacial expansion from multiple southern refugia [57].

We propose to treat the taxa of the complex as subspecies of *Lotus dorycnium* L. Presumed recent and more ancient hybridization apparently played an important role in the formation of the up-to-date pattern of genetic variability of the *L. dorycnium* complex and made it difficult to establish the ancestor (or ancestors) of this group.

## Figures and Tables

**Table 1:** Morphological characteristics of subspecies of *Lotus dorycnium* s.l. [1,9,15,17,19,20].

Characters	ssp. *herbaceus*	ssp. *gracilis*	ssp. *fulgurans*	ssp. *dorycnium*	ssp. *germanicus*	ssp. *anatolicus*	ssp. *haussknechtii*
Life history	Perennial herb, suffrutescent plant or small shrub	Perennial herb	Thorny shrub. Thorns are formed at the ends of the shoots	Perennial herb, suffrutescent plant or small shrub			
Stem length	20–65 cm	30–80 cm	up to 100 cm	10–50 cm	10–50 cm	20–35 cm	30–60 cm
Pubescence	sparse with patent long and somewhat curved hairs	appressed, ±sericeous	appressed, sericeous	appressed	appressed	dense with subpatent long hairs	dense sericeous with appressed short hairs
Leaflet shape	oblong-obovate	linear-oblanceolate to linear	obovate-spathulate	linear-oblanceolate	oblong-obovate	oblong-obovate	oblong-obovate
Leaflet size	4–20 × 2–6 mm	10–20 × 2–4 mm	3.5–7.5 × 1.2–2.3 mm	6–12 × 2–3 mm	(8-)10–20 × 2–4 mm	6–18 × 1–4 mm	8–20 × 1.5–5 mm
Number of flowers per umbel	12–30	12–20	1–4	5–15		4–15	
Peduncles	long		short, up to 0.5 mm	long			
Flower length	3–5 mm		3.2–5.5 mm	4–6(-7) mm			
Pedicels	as long as or longer than calyx tube	usually longer than calyx tube	usually shorter than calyx tube				
Calyx teeth	^1^/_3_–^1^/_2_ (^2^/_3_) length of tube	as long as tube	shorter than tube			^1^/_2_–^3^/_4_ length of tube	

**Table 2:** Taxa, sample code, voucher information, and GenBank accession numbers of *Lotus dorycnium* complex specimens used in molecular and morphological analyses. Herbarium codes according to Index Herbariorum. New sequences indicated by an asterisk (GenBank accession numbers for *rps16* will be added to the final version of the article.).

Sample Code: VOUCHER information (Herbarium Code); Coordinates. Underlined Sample Codes Indicate That the Sample Was Included in the Morphometric Analysis.	ITS	*trn*L-F	*rps*16	*psb*A-*trn*H
*Lotus dorycnium* ssp. *anatolicus*				
1127: Turkey, Çubuk II Barajı, 28.VI.1982, *F. Demircioğlu 1127* (GAZI); 40.0045 N, 32.9329 E	OL688389 *	OL697810 *	OL988837 *	OL753485 *
1443: Turkey, A4 Ankara, Çubuk, Ovacık-Saraycık Köyleri Hallayik pinaiimuk, 03.VIII.1992, *E. Dundar 1443* (GAZI); 40.3047 N, 32.9616 E	OL688390 *	OL697811 *	OL988838 *	OL753486 *
1578: Turkey, A4 Ankara: Kızılcahamam, Soğuksu Milli Parki, Kaya Tepe civari, 10.VI.1990, *O. Eyuboglu 1578* (GAZI); 40.4686 N, 32.6302 E	OL688391 *	OL697812 *	OL988839 *	OL753487 *
1591: Turkey, A4 Çankırı, Atkaracalar, Dumanli Dagi, 09.VII.1992, *Ahmet Duran 1591* (GAZI); 40.7589 N, 33.1222 E	OL688392 *	OL697813 *	OL988840 *	OL753488 *
5885: Turkey, A4 Ankara: Kızılcahamam, 01.VIII.1991, *M. Vural 5885* (GAZI); 40.4703 N, 32.6509 E	OL688393 *	OL697814 *	OL988841 *	OL753489 *
Donmez: Turkey, A4 Kırıkkale: Koҫubaba kasabasi, bağlar yöresi, bozkır, 16.VI.1990, *A. A. Dönmez s.n.* (GAZI); 40.0835 N, 33.8789 E	OL688394 *	OL697815 *	OL988842 *	OL753490 *
***Lotus dorycnium* ssp. *dorycnium***				
D2: Portugal, prov. Trás-os-Montos, Mogadouro, 25.V.1988, *R. Auriault 14166* (H 1657556); 41.3384 N, 6.7202 W	KT250860	MK751661	KT262882	KT262812
D3: Spain, Valencia, Algar, 18.IV.1995, *J. Riera, J. Güemes & E. Estrelles 17073* (H); 39.7808 N, 0.3679 W	KT250862	MK751662	KT262884	KT262814
D7: France, Alpes-Maritimes, Blausasc, 14.V.1977, *A. Charpin & P. Hainard 9350* (H 1456976); 43.8050 N, 7.3634 E	KT250861	MK751660	KT262883	KT262813
PENT03: Spain, Cantabria, Brazomar, en Castro Urdiales, 02.VI.1996, *E. Loriente s.n.* (MA 658686); 43.3745 N, 3.2123 W	OL688395 *	OL697816 *	OL988854 *	OL753502 *
PENT04: Spain, Cantabria, Valle de Bedoya, Cillorigo, 31.VII.1986, *E. Loriente s.n.* (MA 658693); 43.1795 N, 4.5659 W	OL688396 *	OL697817 *	OL988855 *	OL753503 *
PENT06: France, MIDI-Pyrenees, Haute-Garonne, Puymarium, 11.IX.1992, *F.I. van Nek 1042* (P 00851078); 43.3667 N, 0.7500 E	OL688397 *	OL697818 *	OL988856 *	OL753504 *
PENT09: Algeria, Saharan Atlas, near the village Zenina, road to Charet, 10.VII.1968, *V.P. Bochantsev 758* (LE); 34.4585 N, 2.5300 E	OL688398 *	OL697819 *	OL988857 *	OL753505 *
PENT10: Algeria, Saharan Atlas, W of Djelfa, 22.I.1968, *L.E. Rodin* et al. *167* (LE); 34.6729 N, 3.1851 E	OL688399 *	OL697820 *	OL988858 *	OL753506 *
Sp2: Spain, Burgos, on the road from Lerma to Barriosuso, 18 km from Lerma, 07.VI.2018, *T. Kramina & L. Koppel s.n.* (MW); 41.9687 N, 3.5742 W	OL688400 *	OL697821 *	OL988859 *	OL753507 *
***Lotus dorycnium* ssp. *dorycnium* × *L. d.* ssp. *gracilis***				
Fr3: France, Arles, Camargue, bord de canal entre Gageron et Villeneuve, 06.X.1978, *J. & A. Raynal 20915* (P 03031137); 43.5932 N, 4.5945 E	OL688401 *	OL697822 *	OL988860 *	OL753508 *
PENT07: France, MIDI-Pyrenees, Haute-Garonne, road between Peguikhan and Mondilhan, near Busquet-Bas, 01.VI.1993, *F.I. van Nek 1622* (P 00851079); 43.3000 N, 0.7000 E	OL688402 *	OL697823 *	OL988861 *	OL753509 *
PENT08: France, Haute Garonne, Observation Point at St. Felix on N622 to Revel, 04.VII.1980, *Verdcourt & Wilmot-Dear 5358* (P 03615698); 43.4483 N, 1.8905 E	OL688403 *	OL697824 *	OL988862 *	OL753510 *
***Lotus dorycnium* ssp. *haussknechtii***				
2232: Turkey, C6 Kahramanmaraş: Engizeh Dağı, Aksu mahallesi sevresi, 1100 m, 05.VII.1986, *H. Duman & F. Karaveliogulcar 2232* (GAZI); 37.5718 N, 36.9198 E	OL688420 *	OL697840 *	OL988849 *	OL753497 *
4980: Turkey, C3 Antalya: Manavgat-Akseki 10 km, 28.VI.1993, *H. Duman & F. Karavelioğulları 4980* (GAZI); 37.0465 N, 31.7903 E	OL688421 *	OL697841 *	OL988850 *	OL753498 *
7073: Turkey, C5 Adana: Pozantı, 1570 m, 17.VII.1995, *Z. Aytaç & V.N. Adıgüzel 7073* (GAZI); 37.4276 N, 34.8768 E	OL688422 *	OL697842 *	OL988851 *	OL753499 *
9391: Turkey, C3 Antalya, Türkbaş Yaylası-Mahmut Seydi Köyü arası, maki, 10.VI.1993, *Tuna Ekim 9391* (GAZI); 36.6344 N, 32.0242 E	OL688423 *	OL697843 *	OL988852 *	OL753500 *
PENT11: Syria, Mont Amanus, region d’Hasan, VII.1908, *M. Haradjian s.n.* (LE); 36.7508 N, 36.3330 E	OL688424 *	OL697844 *	OL988853 *	OL753501 *
***Lotus dorycnium* ssp. *fulgurans***				
937: United Kingdom, Cultivated at Royal Botanic Gardens, Kew, 2010: origin Spain, Balearic Is.	KT250865	MF314954	KT262887	KT262817
FULG1: Spain, Cabo de Formentor, Baleares, Mallorca, 23.V.1977, *P. Auquier, J. Duvigneaud 77 E 388* (P 03062315); 39.9502 N, 3.1955 E	OL688434 *	OL697856 *	OL988863 *	OL753512 *
***Lotus dorycnium* ssp. *germanicus***				
13: Hungary, Budapest, Sashegy, steppefied meadow on the slope, 23.X.2005, *I. Illarionova 13* (LE); 47.4820 N, 19.0189 E	OL688404 *	OL697825 *	OL988864 *	OL753513 *
131: Slovakia austro-orientalis, distr. Roznava: in declivi cartiensi australi infra arcem Turnansky hrad prope vicum Turna n. Bodvou, c.350 m, 19.VII.1962, *J. Ujcik 131* (LE); 48.6016 N, 20.8765 E	OL688405 *	OL697826 *	OL988865 *	OL753514 *
13472: Italie: Collines au Nord de Rupingrande 350–400 m, 30.V.1976, *M.T. Misset 13472* (LE); 45.7215 N, 13.7875 E	OL688406 *	OL697827 *	OL988866 *	OL753515 *
34293: Slovenija, Polhograjsko hribovje: In pratis prope Govejek supra vicum Medvode. Solo dolom. 800 m, 19.VI.1973, *D. Trpin & T. Wraber 9852/3* (LE); 46.1162 N, 14.3457 E	OL688407 *	OL697828 *	OL988867 *	OL753516 *
D1: Slovenia, Polhograjsko Hribovje, prope Govejek, supra vicum Medvode, 19.VI.1973, *D. Trpin & T. Wraber 9852/3* (H 1081128); 46.1159 N, 14.3459 E	KT250868	MK751666	KT262889	KT262819
D4: Germany, Bayern, Oberbayerische Hochebene, n.München, 06.VII.1991, *H. Kalheber 91-0625* (H 1662801); 48.1374 N, 11.5823 E	KT250869	MK751667	KT262890	KT262820
D5: Montenegro, 40 km NNE of Nikšic, Žabljak, *P. Uotila 10652* (H 1033157); 43.1558 N, 19.1235 E	KT250870	MK751668	KT262891	KT262821
GERM2: Croatia, Kneža, 06.VI.1981, *unknown s.n.* (ZA); 42.9712 N, 17.0518 E	OL688408 *	OL697829 *	OL988868 *	OL753517 *
GERM3: Switzerland, Vaud, Réserve de Pupplinge (Borière) [Borière, Pas de la Borière, Alpes Friburgeoises, commune probable: Grandvillard (8 km E)], VII. 1981, *M. Bournérias 240985* (P 03615699); 46.5362 N, 7.1481 E	OL688409 *	OL697830 *	OL988869 *	OL753518 *
Hv1: Croatia, Istarskaya Zhupanya, 16.V.2016, *I. Schanzer, N. Stepanova, A. Fedorova s.n.* (MW); 45.3222 N, 14.1517 E	OL688410 *	OL697831 *	OL988870 *	OL753519 *
***Lotus dorycnium* ssp. *germanicus* × *L. d.* ssp. *dorycnium***				
PENT05: Austria, Northern Burgenland, W-shore of Lake Neusiedl, 18.VI.2007, *T. Barta s.n.* (P 00851081); 47.9506 N, 16.8390 E	OL688412 *	OL697833 *	OL988872 *	OL753521 *
Au2: Austria, Tirol, Inntal: Zirler Berg NW Zirl, 820 m; Hange nape der Strasse, 05.VIII.1980, *D. Podlech 34422* (P 03020864); 47.2848 N, 11.2249 E	OL688411 *	OL697832 *	OL988871 *	OL753520 *
***Lotus dorycnium* ssp. *germanicus* × *L. d.* ssp. *herbaceus***				
GERM1: Croatia, island Krk, in the port Baška nova, 10.VII.1981, *B. Korica s.n.* (ZA); 44.9709 N, 14.7628 E	OL688413 *	OL697834 *	OL988873 *	OL753522 *
***Lotus dorycnium* ssp. *gracilis***				
6: Spain, Valecnia: El Saler south of Valencia, 14.VIII.1965, *S.A. Renvoize 340* (LE); 39.3825 N, 0.3331 W	OL688414 *	OL697835 *	OL988843 *	OL753491 *
D8: France, dép. Pyrénées-Orientales, Canet, 02.VII.1981, *J. Lambinon, R. Renard & L. Smeets 81/287* (H 1542915); 42.7041 N, 3.0223 E	KT250859	MK751682	KT262881	KT262811
GRAC1: Spain, Castellon, Cabanes, P.N. Prat de Cabanes-Torreblanca, 06.IX.2005, *S. Fos 50/05* (MA 774818); 40.1374 N, 0.1651 E	OL688415 *	OL697836 *	OL988844 *	OL753492 *
GRAC2: Spain, Ciudad Real, Daimiel, Tablas de Daimiel, Isla de Algeciras, 21.VII.1992, *S. Cirujano s.n.* (MA 552216); 39.1628 N, 3.6818 W	OL688416 *OL688417 *	OL697837 *	OL988845 *	OL753493 *
GRAC3: Spain, Cuenca, Garcinarro, hacia Huete, pr. Cerros de Mudarra, 810 m, 10.VII.2004, *V.J. Arán & M.J. Tohá 5930* (MA 732859); 40.2004 N, 2.7225 W	OL688418 *	OL697838 *	OL988846 *	OL753494 *
GRAC4: Spain, Granada, Villanueva de las Torres, 789 m, 08.VII.2008, *A. Amor* et al. *4/7* (MA 838410); 37.5577 N, 3.0888 W	OL688419 *	OL697839 *	OL988847 *	OL753495 *
PENT01: Spain, Castellón, Torreblanca (la Plana Alta), pr. Torrenostra, 30.VII.2012, *V.J. Arán 8084* (MA 877350); 40.1946 N, 0.2245 E	MN545714	MN553697	OL988848 *	OL753496 *
***Lotus dorycnium* ssp. *herbaceus***				
132: Slovakia meridionalis: in declivibus prope vicum Horné Turovce haud procul ab oppido Sahy, 24.VI.1958, *J. Nitka 132* (LE); 48.1214 N, 18.9418 E	OL688425 *	OL697845 *	OL988874 *	OL753525 *
1976: Bulgaria, m. Ograzden: in lapidosis herbosis ad pagum Nikudin, distr. Sandanski, 26.VII.1976, *V. Vakov s.n.* (LE); 41.5661 N, 23.0443 E	OL688426 *	OL697846 *	OL988875 *	OL753526 *
98697: Slovenia, Primorsko: In graminosis fruticosis inter Lokavec prope Ajdovscina et Predmeja, cca 490–500 m, 14.VII.1980, *M. Palma & D. Trpin 49/3* (LE); 45.9237 N, 13.8850 E	OL688427 *	OL697847 *	OL988876 *	OL753527 *
Ab2: Abkhazia, Sukhumi highway, roadside, 07.VI.2019, *M. Lysova & S. Polevova Ab2* (MW); 43.0835 N, 40.8876 E	OL688428 *	OL697848 *	OL988877 *	OL753528 *
BL2: Crimea, Laspi, 02.VI.2015, *C*. *Fomichev BL2* (MW); 44.4157 N, 33.7098 E	MN545735	OL697849 *	OL988878 *	OL753529 *
D6: Austria, Steirisches Hügelland, Steiermark, Umgebung von Radkersburg, 7.VII.1976, *H.Mayrhofer & H.Teppner s.n.* (H 1216503); 46.6897 N, 15.9886 E	KT250882	MK751681	KT262898	KT262828
Gu6: Crimea, Gurzuf, 22.XI.2016, *T.E. Kramina Gu6* (MW); 44.5452 N, 34.2647 E	MN545717	OL697850 *	OL988879 *	OL753530 *
HERB1: Croatia, Šibenik, 15.VI.1997, *M. Milović s.n.* (ZA); 43.7376 N, 15.9094 E	OL688429 *	OL697851 *	OL988880 *	OL753531 *
HERB2: Bulgaria, Burgas prov., Bay Cilistar, 03.VII.2017, *D. Lyskov s.n.* (MW); 42.0232 N, 28.0045 E	OL688430 *	OL697852 *	OL988881 *	OL753532 *
HERB3: Austria, Wien, 14 Bezirk, Hohe Wand-Wiese bei Vordenhainbach, 280–360 m, 26.VII.2004, *Thomas Barta 2004-317* (MA 759930); 48.2298 N, 16.2025 E	OL688431 *	OL697853 *	OL988882 *	OL753533 *
HERB4: Greece, Peloponnesus, Nom. Messinia, Ep. Kalamata, Taijetos Pass between Tripi and Artemisio, 1200–1350 m, 10.VI.1997, *G. Kamari* et al. *s.n.* (MA 871895); 37.0667 N, 22.2667 E	OL688432 *	OL697854 *	OL988883 *	OL753534 *
Kolakovsky: Russia, Krasnodar Krai, Tuapse, 14.VI.1957, *A. Kolakovsky s.n.* (LE); 44.0931 N, 39.0885 E	OL688433 *	OL697855 *	OL988884 *	OL753535 *
Mm1: Crimea, Malyy Mayak, 12.VI.2017, *T.E. Kramina & O.V. Yurtseva Mm1* (MW); 44.6120 N, 34.3594 E	MN545721	MN553698	OL988885 *	OL753536 *
Sh1: Crimea, Shchebetovka, 15.VI.2017, *T.E. Kramina & O.V. Yurtseva Sh1* (MW); 44.9338 N, 35.1458 E	MN545728	MN553701	OL988886 *	OL753537 *
So1: Krasodar krai, Sochi, between Volkonskaya and Soloniki, 05.VI.2017, *M.V. Kuturova So1* (MW); 43.8736 N, 39.3772 E	MN545730	MN553702	OL988887 *	OL753538 *
Tr3: Turkey, Istanbul, 5 km N of Karacaköy, 26.V.2019, *M. Lysova & T. Kramina Tr3* (MW); 41.4503 N, 28.3836 E	OL620157 *	OL624881 *	OL753482 *	OL753539 *
***Lotus dorycnium* ssp. *herbaceus* × *L. d.* ssp. *germanicus***				
Gc2: Greece, Kerkyra, Benitses, 25.VIII.2018, *D.D. Sokoloff Gc2* (MW); 39.5378 N, 19.9117 E	OL620158 *	OL624882 *	OL753483 *	OL753541 *
PENT02: Greece, Peloponnesus, Nom. Messinia, Ep. Kalamata, 6–8 km NE of Ano Amfia, 14.VI.1995, *G. Kamari* et al. *2514* (MA 871946); 37.1389 N, 22.1250 E	MN545713	MN553696	OL988888 *	OL753540 *

**Table 3:** Basic characteristics of variation of plastid markers in geographical groups of *Lotus dorycnium* s.l. and *L. hirsutus*.

	*Lotus dorycnium* Complex			*Lotus hirsutus*	
	Western Group	Eastern Group	Turkish Group *	Western Group	Eastern Group
Number of sequences	19	30	9	8	13
Number of haplotypes	14	25	6	8	10
Number of sites	1993	1999	1985	1990	1986
Invariable sites	1924	1887	1965	1944	1954
Variable (polymorphic) sites:	47 (2.36%)	64 (3.2%)	12 (0.6%)	33 (1.66%)	21 (1.06%)
Singleton variable sites	16	42	7	27	11
Parsimony informative sites	31 (1.56%)	22 (1.1%)	5 (0.25%)	6 (0.3%)	10 (0.5%)
Haplotype diversity Hd	0.953	0.986	0.889	1.000	0.949
Nucleotide diversity Pi	0.00571	0.00432	0.00191	0.00482	0.00305
Mismatch distribution	Multimodal	Unimodal	Multimodal	Multimodal	Multimodal

* Excluding hybrid specimens 4980 and 9391.

## Data Availability

Data is contained within the article or supplementary material.

## Supplementary Materials

The following supporting information can be downloaded at: https://www.mdpi.com/article/10.3390/plants11030410/s1, Figure S1: Maximum Likelihood phylogenetic tree of the genus *Lotus* based on nrITS dataset (constructed using IQ-tree software). Figure S2: Maximum likelihood phylogenetic tree of the genus *Lotus* based on the combined plastid dataset (constructed using IQ-tree software). Figure S3: Maximum likelihood phylogenetic tree of the genus *Lotus* based on nrITS dataset (constructed using RAxML software). Figure S4: Maximum likelihood phylogenetic tree of the genus *Lotus* based on the combined plastid dataset (constructed using RAxML software). Dataset S1: nrITS dataset. Dataset S2: combined plastid dataset (*trn*L-F, *rps*16 and *psb*A-trnH).

## Appendix A

Taxa, sample code, locality, voucher information (herbarium code) of *Lotus, Cytisopsis, Hammatolobium* and *Tripodion* species used in molecular analyses. GenBank accession numbers of sequences, taken from GenBank or newly sequenced in this study, are given for the four markers, ITS, *trn*L-F, *rps*16, *psb*A-*trn*H. New sequences indicated by asterisks.

***Cytisopsis pseudocytisus* (Boiss.) Fertig: 7**, Turkey, C1, Muğla, Datça, Knidos, 29–31.V.1995, A.P. Khokhryakov & M.T. Mazurenko s.n. (MHA), AY325282, MK751647, HM468299, HM468259; ***Hammatolobium kremerianum* (Coss.) C.Muell.: 643**, Morocco, Podlech 51378 (MHA), KT250926, MK751648, KT262933, KT262863; ***Lotus aegaeus* Boiss.: 427**, Turkey, C3, Antalya Korkuteli, Termessos, Büyükkumluca, Çakıllı geçidi, 04.VI.1995, A.P. Khokhryakov & M.T. Mazurenko 1135 (MHA), DQ160276, MK751649, KT262865, KT262794; ***Lotus angustissimus* L.: 472**, Australia, Norfolk Island, introduced, 14.X.1999, B.M. Waterhouse 5510 (NSW), DQ166243, MF158217, KT262868, KT262798; ***L. axilliflorus* (Hub.-Mor.) D.D. Sokoloff: 5089**, Turkey, C2 Burdur, Yeşilova, Salda gölü, 12.VIII.1993, H. Duman, Z. Aytaç and Dönmez 5089 (GAZI), MW412842, MW470873, MW498319, OL753484 *; **941**, Turkey, Duman et al. 5089 (E), KT250852, MN553691, KT262869, KT262799; ***Lotus broussonetii* Choisy ex Ser.: 21**, Cultivated at Royal Botanic Gardens, Kew: introduced from Canary Is., DQ160278, MK751653, KT262872, KT262802; ***Lotus castellanus* Boiss. & Reut.: 471**, Spain, Segura Zubizarreta 38111 (MHA), DQ166238, MF158215, KT262873, KT262803; ***Lotus conimbricensis* Brot.: 485**, Spain, Badajoz, Almendral, 27.IV.1966, Segura Zubizarreta 960 (Z), FJ411114, MF158231, KT262874, KT262804; ***Lotus corniculatus* L., L7**, Russia, Moscow prov., Lutsino, 03.VII.2008, Kramina 74-7 (MW), JF784200 & JF784201, MW470874, KT262876, KT262806; ***Lotus creticus* L.: 501**, Cultivated in Australia from seeds collected in Azores Is., Sandral SA39213 (MW), FJ938296, OL697808 *, KT262877, KT262807; ***Lotus cytisoides* L.: 425**, Cyprus, Seregin & Sokoloff 280 (MW), DQ166241, DQ160280, OL697809 *, KT262878, KT262808; ***Lotus discolor* E. Mey.: 444**, Cameroon, S. Lisowski B-3330 (BR), DQ160288, MK751659, KT262880, KT262810; ***Lotus edulis* L.: 623**, Cyprus, 10 km to W from Limassol, 13.III.2004, Seregin & Sokoloff A-280 (MW), KT250863, MK751663, KT262885, KT262815; ***Lotus eriophthalmus* Webb & Berthel.: ERIO**, Spain, Tenerife, Cultivated at Botany Dept. of University of La Laguna, 11.V.1984, A. Gharpin & M. del Asco 185745 (MA 318437), MW412843, MW470875, MW498320, OL753511 *; ***Lotus glinoides* Del.: 461**, Egypt, 7.V.1962, Bochantsev s.n. (LE), DQ166220, MK751677, KT262892, KT262822; ***Lotus graecus* L.: 2459**, Turkey, B3 Kütahya, Dumlupınar, Gökdağ, Akdene mevkii, 22.VII.1983, M. Vural & F. Maluen 2459 (GAZI), MW412844, MW470876, MW498321, OL753523 *; **Ca1**, Crimea, Vinogradnoye, mount Castell, 13.VI.2017, T.E. Kramina Ca1 (MW), MN545698, MN553692, MW498325, OL753524 *; **D10**, Greece, East Macedonia, Thasos, Glifada, 18.V.1986, T. Raithalme s.n. (H), KT250877, MK751678, KT262894, KT262824; **D9**, Turkey, A3, Bolu, Düzce-Akçakoca, 24.V.1990, R. Lampinen 7871 (H), KT250876, MK751679, KT262893, KT262823; ***Lotus halophilus* Boiss. & Spruner: 431**, Greece, Karpathos, Pigadia, 19.IV.1984, Th.Raus 9307 (MHA), KT250879, MK751680, KT262896, KT262826; ***Lotus hirsutus* L.: 03052322**, Greece, Macedonia, Kalithea, 00.IV.1995, G. Van Buggenhout 17072 (P 03052322), MW412853, MW470888, MW498337, OL753542 *; **03052323**, Spain, Prov. Teruel, Mosqueruella, 24.V.1992, C. Fabregat & S. López s.n. (P 03052323), MW412854, MW470889, MW498338, OL753543 *; **03052351**, France, Aude, Massif de la Clape, 16.V.1975, B. de Retz 71072 (P 03052351), MW412855, MW470891, MW498340, OL753544 *; **1**, Spain, prov. Teruel, Mosqueruella, 24.V.1992, C. Fabregat & S. López s.n. (MHA), MW412856, MW470892, MW498341, OL753545 *; **1841**, Turkey, C2 Muğla, Marmaris, Bağli Tepe civarı, 27.VI.1997, H. Sağban 1841 (GAZI), MW412857, MW470893, MW498342, OL753546 *; **2058**, Turkey, C5 Adana, Karataş, Yumurtalık Lagünü, 19.IV.1998, H. Sağban 2058 (GAZI), MW412858, MW470894, MW498343, OL753547 *; **343816**, Spain, Gerona, Girones, Canet d’Adri, 16.V.1986, E. Castells & J. Pedrol s.n. (MA 343816-2), MW412859, MW470895, MW498344, OL753548 *; **4**, Montenegro, 30 km of Titograd, NE of Petrovac, 19.VI.1971, P. Uotila 10633 (MHA), MW412860, MW412861, MW470896, MW498345, OL753549 *; **609**, Spain, near Barcelona, July 2006, A.S. Beer & S.S.Beer s.n. (MW), KT250886, MK751683, KT262902, OL753550 *; **626236**, Spain, prov. Huesca, Rodellar, Sierra de Rufas, 22.V.1970, P.Montserrat s.n. (MA 626236), MW412862, MW470897, MW498346, OL753551 *; **7**, Turkey, C1 Aydın, Menderes Nehri, Bafa Gölü, 28.V.1995, A.P. Khokhryakov & M.T. Mazurenko s.n. (MHA), MW412863, MW470898, MW498347, OL753552 *; **D11**, Turkey, A1 Çanakkale, Yalova-Eceabat, 15.V.1990, R. Lampinen 7355 (H), KT250883, MN553705, KT262899, KT262829; **D12**, Greece, East Macedonia, Thasos, Glifada, 19.V.1986, T. Raithalme s.n. (H), KT250885, MK751684, KT262901, KT262831; **D13**, Croatia, Korcula island, SW of Pupnat, 23.VI.1971, L. Hämet-Ahti 2225 (H), KT250884, MK751685, KT262900, KT262830; **GC3**, Greece, Kerkyra, Benitses, 25.VIII.2018, D.D. Sokoloff s.n. (MW), MW412864, MW470899, MW498348, OL753553 *; **HIRS1**, Spain, Madrid, Alcala de Henares, 07.VII.1996, E. Soberino Vesperinas s.n. (MA 582050), MN545736, MN553706, MW498349, OL753554 *; **HIRS2**, Italia, Sicilia, Ragusa, 09.VI.2000, Alvares et al. IA 1784 (MA 645120), MN545737, MN553707, MW498350, OL753555 *; **HIRS3**, Croatia, Lokrum, 15.V.1977, S. Heéimovié s.n. (ZA), MW412865, MW470900, MW498351, OL753556 *; **HIRS4**, Croatia, island Biševo, 25.VII.1981, B. Korica s.n. (ZA), MW412866, MW470901, MW498352, OL753557 *; **HIRS5**, Croatia, Zakovae (Šibenik), 29.V.1997, M. Milović s.n. (ZA), MW412867, MW470902, MW498353, OL753558 *; **Sp4**, Portugal, Algarve, Vila do Bispo, 06.VI.2001, L. Medina, S. Nisa, M. Pardo de Santayana s.n. (MA), MW412870, MW470905, MW498356, OL753559 *; ***Lotus laricus* Rech.f., Aellen & Esfand.: 455**, Abu Dhabi, Abu Dhabi Island, Al Mushrif Palaca, 04.V.1982, R.A.Western 275 (E), DQ166233, MK751687, KT262906, KT262836; ***Lotus maculatus* Breitf.: 958**, Canary Is. (cult.), Tenerif. Municipio de la Orotava, Puerto de la Cruz, 14.IV.2000, H. Väre 10894 & H. Kaipiainen (H 1702795), KT250890, MK751688, KT262907, KT262837; ***Lotus ononopsis* Balf. f.: 453**, Yemen, Muqadrihon Pass, c. 10 km SW of Hadiboh, 26.I.1990, A.G. Miller et al. 10097 (E), DQ166219, MK751690, KT262909, KT262839; ***Lotus parviflorus* Desf.: 469**, Spain, Talavera-de-la-Reina, 09.V.1987, Segura Zubizarreta 34.567 (MHA), DQ166230, MF314955, MW498357, OL753560 *; ***Lotus pedunculatus* Cav.: 437**, Spain, Soria, Santa Inés, 18.VII.1972, Segura Zubizarreta s.n. (LE), DQ166222, MF158224, KT262910, KT262840; ***Lotus rectus* L.: 401**, Lebanon, on the bank of the Nahr el Kalb, 05.VI.1959, T.D. Maitland 401 (LE), MW412874, MW470909, MW498361, OL753561 *; **955**, Crete, Retimno, 00.VIII.2012, Sokoloff s.n. (MW), KT250902, MW470912, KT262915, KT262845; **REC1**, Spain, Alicante, Rio Guadalest, 02.VII.1958, A.Rigual s.n. (MA 373077), MK780164, MK751693, MW498364, OL753562 *; ***Lotus sanguineus* (Vural) D.D. Sokoloff: 940**, Turkey, C4 Konya, 00.00.1981, M. Vural 1976 (E), KT250904, MN553710, KT262916, KT262846; ***Lotus spectabilis* Choisy ex Ser.: SPEC**, Spain, Tenerife, Güimar, 00.VIII.1977, A. Santos-Ricardo 5124 (MA 839030), MW412881, MW470917, MW498370, OL753563 *; ***Lotus strictus* Fisch. & C.A.Mey.: 3845**, Turkey, B4, Tuz gölü, Aksaray-Eşmekaya sazlığı, 13.VII.1997, M. Aydoğdu 3845 (ANK), MW412882, MW470918, MW498371, OL753564 *; **413**, Russia, Altai Krai, Mikhaylovsky distr. 18.IX.2003, Korolyuk s.n. (MW), DQ160286, MF158210, KT262923, KT262853; **923**, Kazakhstan, Pavlodar Prov., Kanonerka, 00.00.1956, I. Povalyaeva s.n. (MW), KT250914, MF158211, KT262924, KT262854; ***Lotus subbiflorus* Lag.: 470**, Italy, Lazio, Pianura Pontina, 15.06.1991, M. Iberite 15222 (MHA), DQ166231, MF158212, KT262925, KT262855; ***Lotus tetragonolobus* L.: 624**, Cyprus, to E from Limassol, Amathus, 08.III.2004, A. Seregin & al. A-110 (MW), HM468334, MK751696, KT262927, KT262857; ***Lotus villicarpus* Andr. (syn. *L. eriosolen* (Maire) Mader et Podlech): 414**, Morocco, prov. Ourzazate, 06.IV.1995, D.Podlech 52619 (M), DQ160281, MK751664, KT262886, KT262816; ***Tripodion tetraphyllum* (L.) Fourr.: 625**, Cyprus, 7.5 km to N from Limassol, 11.III.2004, A. Seregin & D. Sokoloff A-240 (MW), HM468340, MK751698, HM468314, HM468274.

## Appendix B

Taxa, sample code and voucher information of *Lotus dorycnium* complex specimens used in morphological analyses only.

***Lotus dorycnium* ssp. *anatolicus*: 11373**, Turkey, T. Baytop 11373 (ISTE); **25568**, Turkey, A. Baytop and E. Tuzlacı 25568 (ISTE); **34997**, Turkey, A. Baytop and K. Alpınar 34997 (ISTE); **37924**, Turkey, K. Alpınar 37924 (ISTE); **4208**, Turkey, M. Vural 4208 (GAZI); **4291**, Turkey, J. Bornmüller 4291 (LE); **478**, Lebanon, J. Bornmüller 478 (LE); **52370**, Turkey, K. Alpınar 52 370 (ISTE); **94024**, Turkey, B. Yıldız 5789 and N. Çelik, 94024 (ISTE); **967**, Turkey, M. Vural 967 (GAZI); ***Lotus dorycnium* ssp. *anatolicus* × ssp. *haussknechtii***, **1916**, Turkey, B. Shishkin s.n., 03.07.1916 (LE); **2793**, Turkey, C. Haussknecht 2793 (LE); ***Lotus dorycnium* ssp. *germanicus***, **1908**, Croatia, A. De Degen s.n., 25.06.1908 (LE); **1911**, [Czech Republic], Moravia, H. Laus s.n., VII.1911 (LE); **1917**, Hungary, A. De Degen s.n., 17.06.1917 (LE); **22402**, Turkey, A. Baytop 22.402 (ISTE); **63997**, Turkey, T. Cerit 46, 63 997 (ISTE); **1905**, Croatia, A. De Degen s.n., 04.07.1905 (LE); ***Lotus dorycnium* ssp. *gracilis*, 787258**, Spain, S. Fos and M.A. Codoñer 7/89 (MA); ***Lotus dorycnium* ssp. *haussknechtii***, **26706**, Turkey, H. Demiriz 26.706 (ISTE); **73323**, Turkey, A.J. Byfield and D. Pearman [B 2581], 73323 (ISTE); **77992**, Turkey, M. Keskin 77 992 (ISTE); **9732**, Turkey, A. Baytop et al. 9732 (ISTE); **2627**, Turkey, A.Duran 2627 (GAZI); ***Lotus dorycnium* ssp. *herbaceus*, 25036**, Turkey, G. Ertem 25036 (ISTE); **25480**, Turkey, A. Baytop and E. Tuzlacı 25480 (ISTE); **30223**, Turkey, A. Baytop and E. Tuzlacı 30223 (ISTE); **3537**, Turkey, A. Berk and T. Baytop 3537 (ISTE); **38443**, Turkey, K. Alpınar 38 443 (ISTE); **63996**, Turkey, T. Cerit 44, 63996 (ISTE); **77656**, Turkey, M. Keskin 77656 (ISTE); **80902**, Turkey, Ş. Kültür and N. Sadıkoğlu 80902 (ISTE); **92120**, Turkey, E. Akalın and H. Demirci 92120 (ISTE); **9395**, Turkey, J.Bornmüller 9395 (LE); **96346**, Turkey, N. Güler, H. Ersoy 96346 (ISTE); **BL1**, Crimea, C. Fomichev BL1 (MW); **Gu9, Gu10**, Crimea, T. Kramina Gu9, Gu10 (MW); **Mm2-Mm5**, Crimea, T.Kramina and O.Yurtseva Mm2, Mm3, Mm4, Mm5 (MW); **Ni1-Ni2**, Crimea, T. Kramina Ni1, Ni2 (MW); **Sh2**,Crimea, T. Kramina and O. Yurtseva Sh2 (MW); **So7**, Russia, Caucasus, M. Kuturova So7 (MW); **VS1**, Crimea, P. Karpunina VS1 (MW); **Typus-D**, Crimea, Ledebour (LE, Typus of *Dorycnium intermedium* Ledeb.); ***Lotus dorycnium* ssp. *dorycnium* × *Lotus dorycnium* ssp. *herbaceus***, **1842**, Italy, Bracht s.n., 1842 (LE); **2-4**, Albania, Kuvaev 2-4 (LE); **Chrtek**, Slovakia, Chrtek and Křísa s.n., 06.10.1974 (LE); ***Lotus dorycnium* ssp. *dorycnium***, **Höpflinger**, Spain, Balearic Islands, F.Höpflinger s.n., 25.05.1976 (MHA); **Sp3**, Spain, T. Kramina and L. Koppel s.n. (MW); ***Lotus dorycnium* ssp. *dorycnium* × *L. dorycnium* ssp. *gracilis***, **14166**, Portugal, R. Auriault 14166 (MHA); **9350**, France, A. Charpin and P. Hainard 13887 (MHA).
