# Supplementary figures and images for: When Morphology and Biogeography Approximate Nuclear ITS but Conflict with Plastid Phylogeny: Phylogeography of the Lotus dorycnium Species Complex (Leguminosae)
Source: Plants (Basel). 2022 Feb 2;11(3):410. doi: 10.3390/plants11030410 (PMC8840542; doi:10.3390/plants11030410)

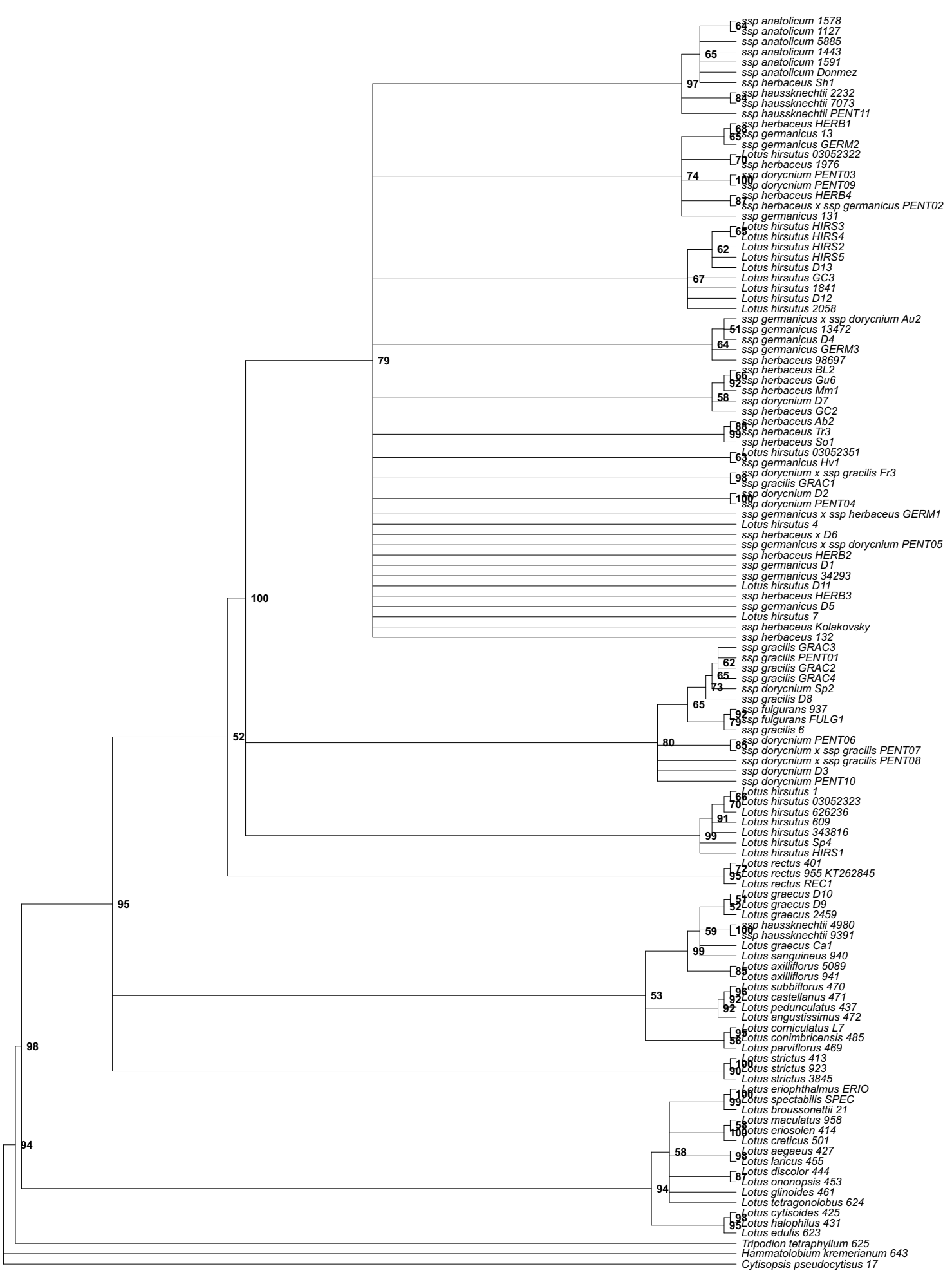

Supplement: Supplementary file 1 [file plants-11-00410-s001.zip › Figure-S4-cpDNA-ML-RAxML.pdf]

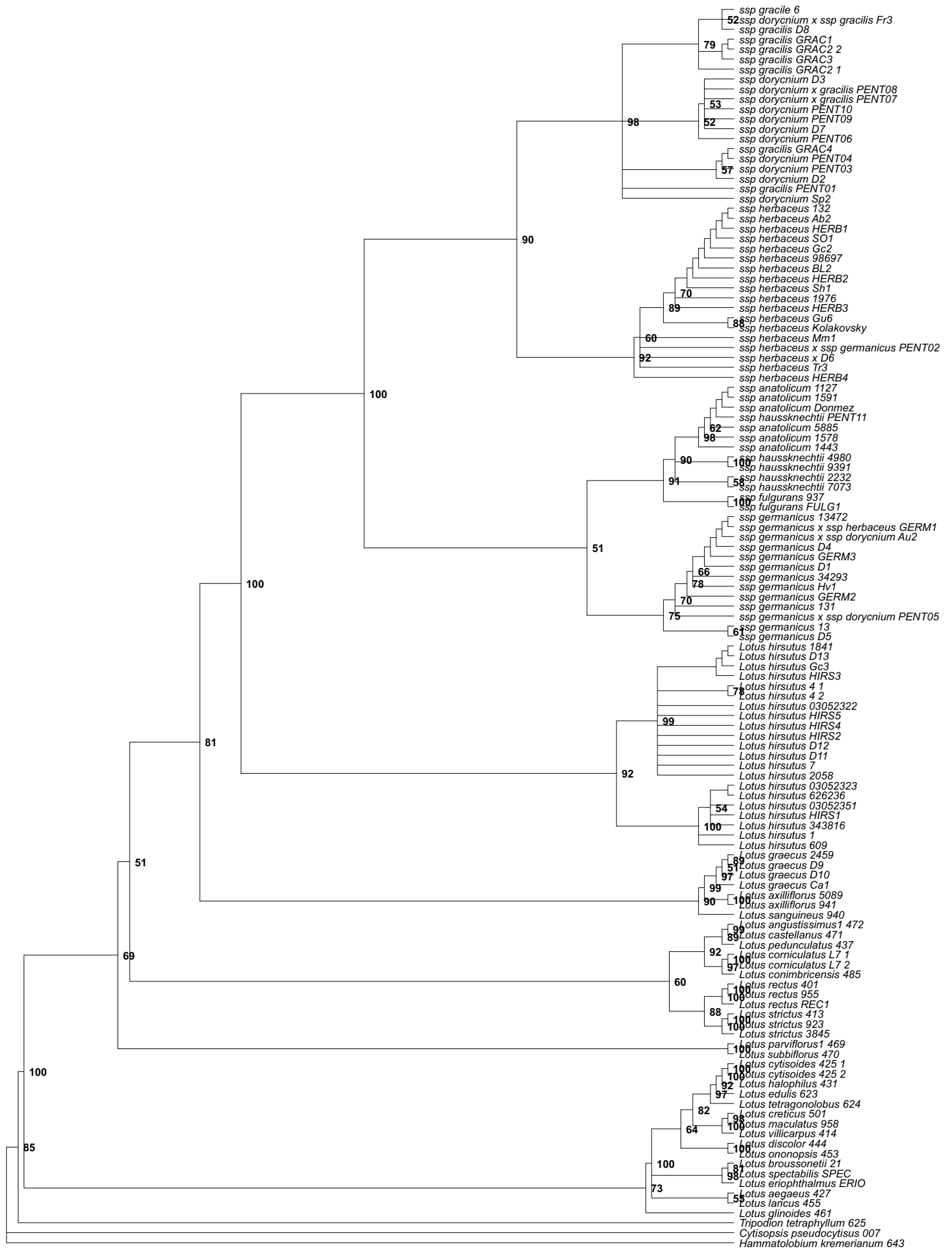

Supplement: Supplementary file 1 [file plants-11-00410-s001.zip › Figure-S1-ML-ITS-2022-01-18.pdf]

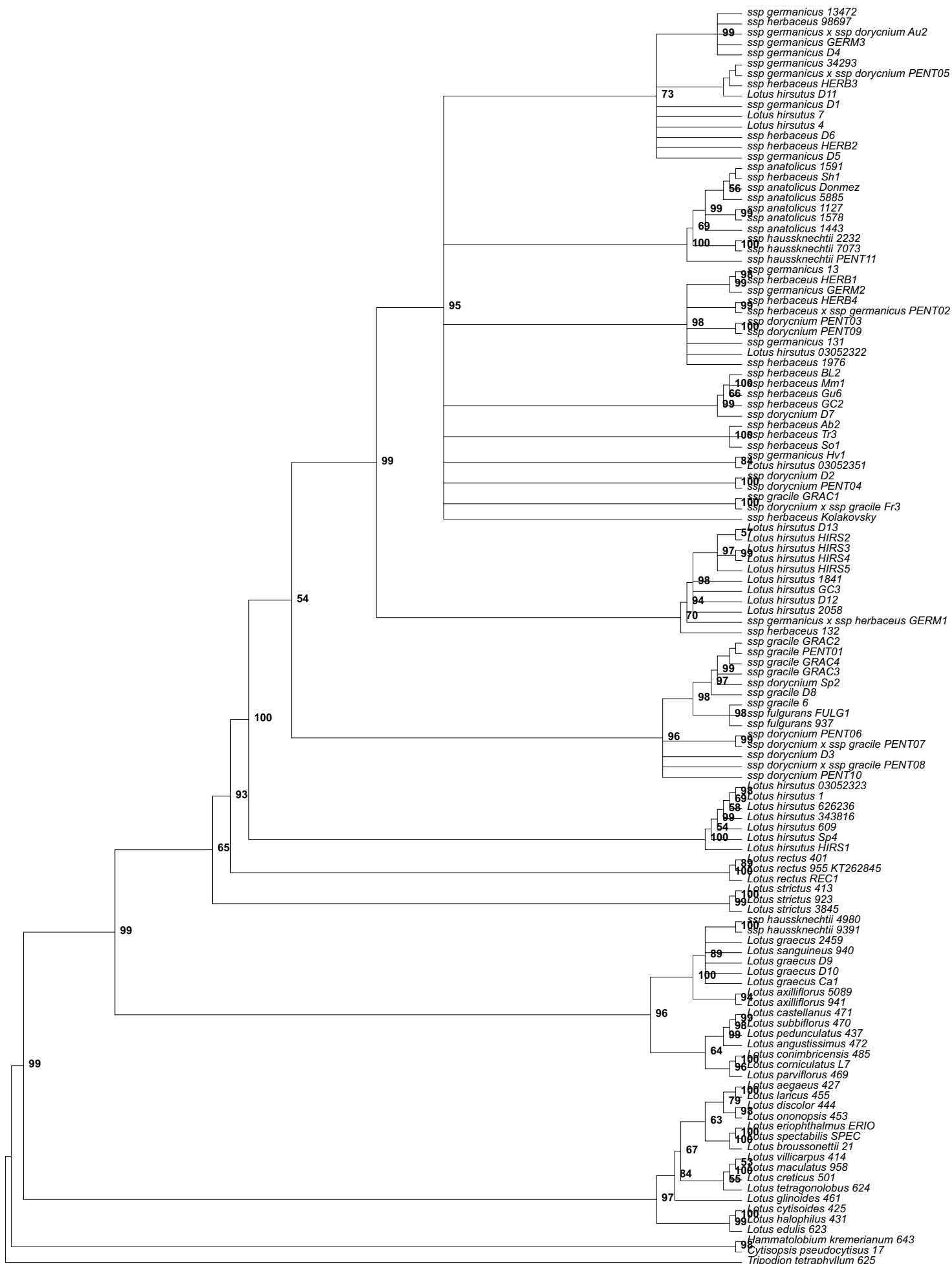

Supplement: Supplementary file 1 [file plants-11-00410-s001.zip › Figure-S2-ML-cpDNA-2022-01-18.pdf]

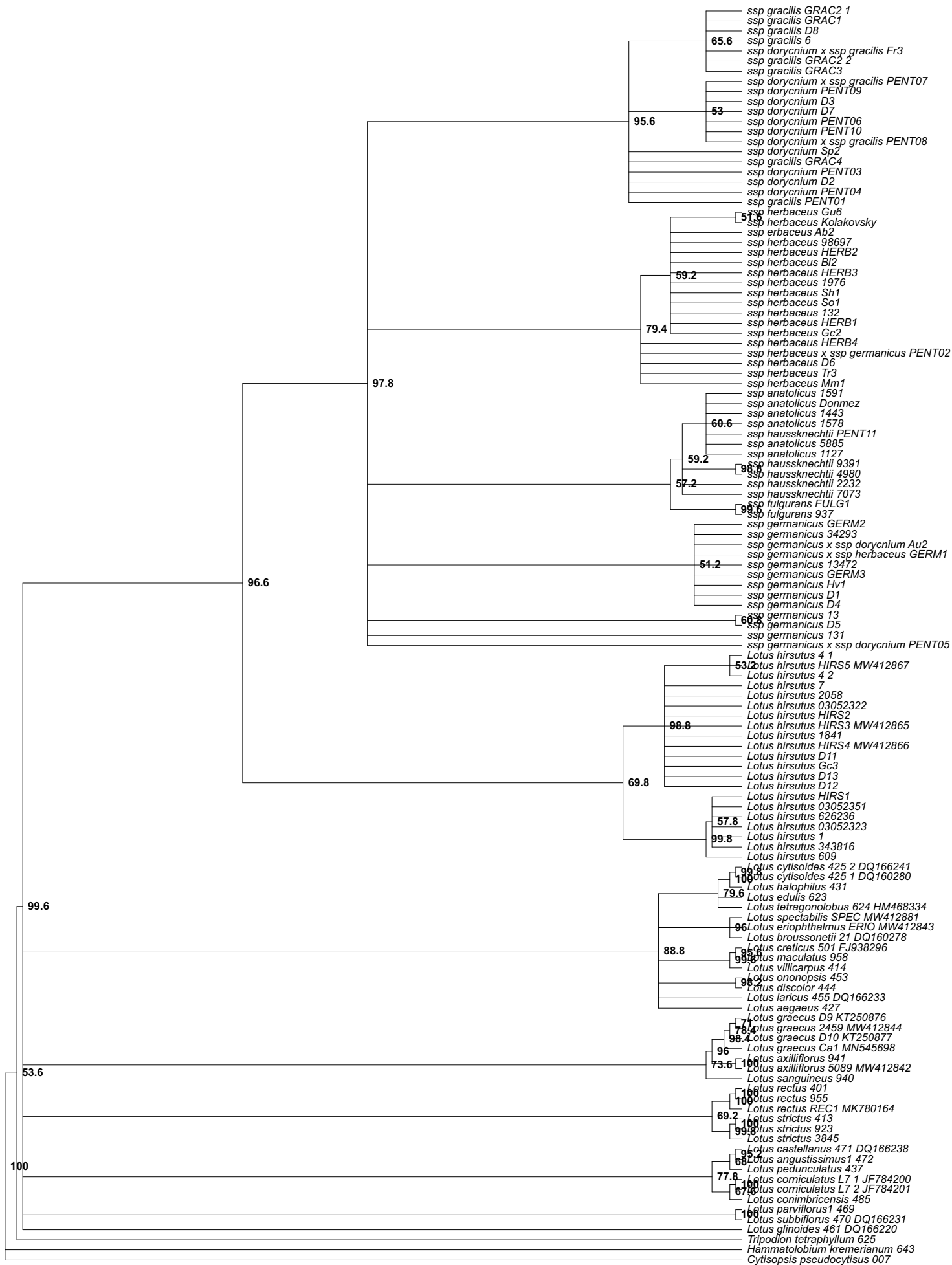

Supplement: Supplementary file 1 [file plants-11-00410-s001.zip › Figure-S3-ITS-ML-RAxML.pdf]
